# Biochemical Characterization of a Kunitz‐Type Protease Inhibitor From *Mimosa regnellii* and Its Effects on Melanoma Cell Viability and Angiogenesis

**DOI:** 10.1002/cbdv.202503760

**Published:** 2026-04-21

**Authors:** Luciana Maria Araújo Rabêlo, Pedro Henrique de Oliveira Cardoso, Leonardo Thiago Duarte Barreto Nobre, Paula Ivani Medeiros dos Santos, Sheyla Varela Lucena, Raphael Paschoal Serquiz, Marcelo Porto Bemquerer, Hugo Alexandre de Oliveira Rocha, Helena Bonciani Nader, Elizeu Antunes dos Santos, Adeliana Silva de Oliveira, Breno Emanuel Farias Frihling, Ludovico Migliolo

**Affiliations:** ^1^ Laboratório De Química e Função De Proteínas Bioativas Centro de Biociências Departamento De Bioquímica Universidade Federal do Rio Grande Do Norte Natal Rio Grande do Norte Brazil; ^2^ Programa de Pós‐Graduação em Ciências Ambientais e Sustentabilidade Agropecuária Universidade Católica Dom Bosco (UCDB) Campo Grande Brazil; ^3^ Laboratório De Purificação de Proteínas e suas Funções Biológicas Programa de Pós‐Graduação em Biotecnologia Universidade Católica Dom Bosco Campo Grande Brazil; ^4^ Departamento De Bioquímica Universidade Federal de São Paulo (UNIFESP) São Paulo São Paulo Brazil; ^5^ Diretoria de Recursos Naturais Programa de Mestrado em Uso Sustentável dos Recursos Naturais Instituto Federal de Ciências e Tecnologia Do Rio Grande Do Norte Natal Rio Grande do Norte Brazil; ^6^ Embrapa Gado de Leite Juiz de Fora Minas Gerais Brazil; ^7^ Laboratório De Biotecnologia de Polímeros Naturais‐BIOPOL Departamento De Bioquímica Universidade Federal do Rio Grande Do Norte Natal Rio Grande do Norte Brazil; ^8^ Laboratório De Proteômica Instituto De Medicina Tropical Do Rio Grande Do Norte Centro de Biociências Universidade Federal do Rio Grande Do Norte Natal Rio Grande do Norte Brazil; ^9^ Laboratório De Química e Função De Proteínas Bioativas Departamento De Bioquímica Centro de Biociências Universidade Federal do Rio Grande Do Norte Natal Rio Grande do Norte Brazil

**Keywords:** angiogenesis, cell migration, melanoma, mitochondrial apoptosis, protease inhibitor

## Abstract

Cancer is a term used to represent more than 100 diseases, including malignant tumors of different localizations. Among these types, cutaneous melanoma is one of the most common types of skin cancer worldwide. Newer, non‐invasive, and more effective forms of treatment are urgently needed. Recently, the cancer membrane has emerged as a novel target for new anticancer drugs. Serine protease inhibitors are researched across all kingdoms; however, the Plantae kingdom is a more sustainable producer of bioactive molecules, including serine protease inhibitors. In this study, a Kunitz trypsin inhibitor (JTI) purified by RP‐HPLC from *Mimosa regnellii* seeds was sequenced, its inhibitory activity quantified, and its inhibition constant determined. It was then tested for its ability to induce cell death via the apoptotic pathway in B16‐F10 mouse melanoma cells. To evaluate its pro‐apoptotic capacity, the inhibitor's effects on reactive oxygen species release, calcium release, cell morphological changes, and the inhibition of angiogenic and migratory activities of rabbit endothelial cells (RaEC) were also assessed in vitro. Tests for toxicity against normal cells were performed, confirming that JTI acts on melanoma cells with an IC_50_ of 0.65 µM. These results suggest that JTI has potential for use in melanoma treatment.

## Graphical abstract

A Kunitz‐type serine protease inhibitor (JTI) purified from *Mimosa regnellii* seeds selectively targets melanoma cells. JTI triggers mitochondrial apoptosis, increases ROS and Ca^2^^+^ levels, arrests the cell cycle, and suppresses migration and angiogenesis without affecting healthy cells. The findings highlight JTI as a promising plant‐derived candidate for innovative melanoma therapies.

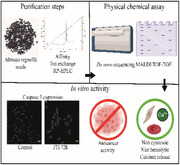

## 1 Introduction

Cancer is commonly used as a synonym for a group of diseases characterized by the uncontrolled growth of cells, caused by alterations in cell growth programs. Risk factors for developing cancer can be hereditary or environmentally induced. These external changes can influence the development of different types of cancer. Cutaneous melanoma, a common type of skin cancer worldwide, originates from melanocytes and is most prevalent in white adults. Similar to many other cancers with environmental etiologies, in this case, UV radiation incidence increases significantly with age, presumably reflecting the lag time between exposure and cancer development [1].

Alterations in signal transduction pathways play a crucial role in regulating the cell cycle and cell survival and are essential for the establishment of all tumor types. Thus, these two pathways are fundamental to the establishment and development of cancer. The vast catalog of cancer cell genotypes reflects six essential alterations in cell physiology that collectively drive malignant growth signals: insensitivity to growth‐inhibitory signals, evasion of programmed cell death (apoptosis), unlimited replicative potential, sustained angiogenesis, tissue invasion, and metastasis [2].

On the other hand, many forms of treatment and prevention have been explored, including the inhibition of molecular targets on the cancer cell membrane surface, such as proteases [3], which are protein enzymes that catalyze the hydrolysis of peptide bonds, resulting in smaller peptides or amino acids [4]. The serine endopeptidases represent a group of protein enzymes well characterized in the literature. Due to the potential hazard of proteolytic enzymes in the environments where they operate, their activities must be controlled. To achieve this, organisms use protease inhibitors as a primary mechanism to regulate proteolytic activities by complexing with and blocking the action of these endopeptidases; thus, the inhibition of serine endopeptidases in cancer cells without deleterious effects represents a promising cancer treatment [3].

A source of serine protease inhibitors with medical application in the treatment of cancer is the kingdom Plantae [5] specially in the Fabaceae family with present broad‐spectrum activity against multiple cancer cell lines with inhibitory such as TIC from *Cajanus cajan* with inhibitory concentration (IC_50_) of 4.2 µM against Caco‐2 cell line [6], CdTI from *Caesalpinia decapetala* with IC_50_ of 0.397 µM against A549 cell line [7]. PDTI, a Fabaceae‐derived Kunitz‐type inhibitor from *Peltophorum dubium* can directly decrease cellular viability and induce caspase‐3‐like mediated apoptosis in Nb2 lymphoma cells [8].

Within the Fabaceae family, the *Mimosa* genus has multiple species which are considered valuable medicinal plants due to their ethnotraditional medicinal use in different parts of the world to treat different illnesses, including cancer [9, 10]. The species *Mimosa regnellii* Benth is widely distributed in South America, especially in endemic areas of Brazil and Argentina [11]; however, studies on its biotechnological properties applied to medicine are virtually nonexistent.

In this work, a potent serine protease inhibitor was purified from *M. regnellii* (Juquiri) seeds using RP‐HPLC. Its enzymatic inhibitory activity, Ki determination, and primary sequence analysis were carried out, followed by biological characterization through its hemolytic activity and cytotoxicity against the B16‐F10 melanoma cell line. This was confirmed by viability assays, cellular proliferation analysis, cell death marker evaluation, changes in mitochondrial membrane potential, and its influence on angiogenic processes and cell migration.

## 2 Results

### 2.1 Purification of Juquiri Trypsin Inhibitor (JTI)

The JTI trypsin inhibitor was purified from the total seed extract, followed by ammonium sulfate precipitation and three chromatographic steps: affinity chromatography, ion exchange chromatography, and RP‐HPLC (Reverse‐phase high‐performance liquid chromatography) C8 column [12, 13]. The 30%–60% (NH_4_)_2_SO_4_ fraction, which showed the highest inhibitory activity against trypsin (82.41 ± 1.17%) (Figure S1) was dialyzed and loaded onto a CNBr‐activated Sepharose 4B trypsin affinity column. The retained peak was further loaded onto a DEAE‐Sephadex ion exchange column, and the eluate with inhibitory activity was obtained.

**FIGURE 1:**
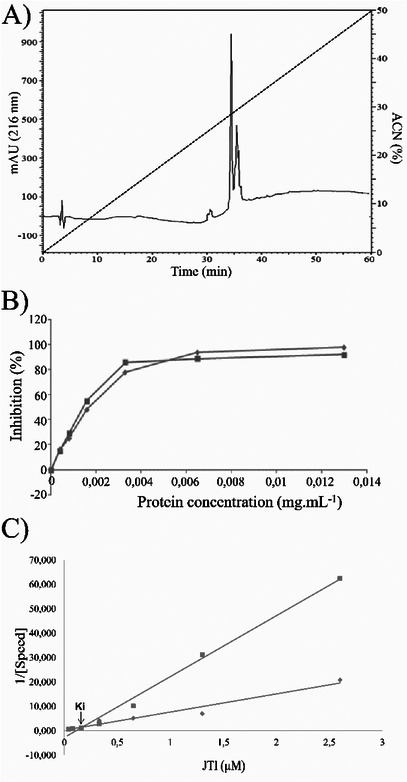
JTI chemical profile. (A) Chromatogram of JTI in high‐performance reverse phase liquid chromatography (HPLC‐RP) in a C8 column. Elution with an increasing linear gradient (0%–100% acetonitrile). (B) Determination of enzymatic IC_50_ JTI. Titration curve using increasing concentrations of JTI: 0.04, 0.08, 0.16, 0.33, 0.65, 1.3, and 2.6 µM and a fixed concentration of trypsin. (C) Determining the value of JTI Ki. The JTI inhibition constant is 1.7 × 10^−6^ M; legend: square—BApNA 0.625 mM; diamond—BApNA 1.25 mM.

After this purification step, some contaminants observed in SDS‐PAGE electrophoresis remained, and a final chromatographic step was performed. Purification of the inhibitor was performed by RP‐HPLC and a chromatographic run, where four peaks were obtained and tested against trypsin. The peak 3 (P3) showed in SDS‐PAGE electrophoresis (Figure S2) represented the purified active inhibitor, named JTI (Figure 1A), that was further dialyzed and lyophilized for the next assays.

The purification pattern through different sequential steps resulted in a 623.8‐fold purification of JTI with a recovery of 10.8% yield (Table 1). The molecular mass of JTI is 17.9 kDa, as estimated by 15% SDS‐PAGE electrophoresis, which showed a single protein band, calculated by linear regression (Figure S3), using standard molecular weight proteins as markers.

**TABLE 1:** Purification table of Juquiri trypsin inhibitor (JTI).

Sample	Volume (mL)	Protein (mg mL^−1^)	Total protein(mg)	Activity (UI mL^−1^)	Specific activity (UI mg^−1^)	Total activity (UI)	Purification (x)	Yield (%)
**CE**	62	1.9627	121.685	11.9	0.1	737.8	1.0	100.0
**F2**	18	1.2053	21.696	12.8	0.6	230.4	6.0	31.2
**AF**	26	0.0093	0.242	8	33.1	208.0	338.5	28.2
**IC**	8	0.0021	0.016	10	609.4	80.0	623.8	10.8

*Note*: CE, crude extract; F2, fraction 2; AF, affinity; IC, DEAE‐Sephadex.

To evaluate the chemical stability of JTI, samples were separately exposed to denaturing (SDS, 10%) and reducing agents (DTT, 25 mM). Electrophoretic analysis on a 15% polyacrylamide gel of the treated samples showed that the chemicals did not promote the appearance of new protein bands, indicating that JTI does not have a structure composed of subunits linked by disulfide bonds or hydrophobic interactions (Figure S2).

### 2.2 Enzyme Inhibitory Activity and Ki Determination Assays

The concentration of JTI, which inhibits 50% of trypsin activity (enzymatic IC_50_), was determined by constructing a titration curve that relates the percentage of inhibition to the trypsin concentration and JTI (Figure 1B). The percentage of inhibition was determined with trypsin activity for each concentration of inhibitor used, and a titration curve was constructed, where the value of enzymatic IC_50_ was determined to be 2 × 10^−4^ mM.

For determining the inhibition mechanism of JTI, a Lineweaver–Burk double reciprocal graph was constructed, performed with varying concentrations of inhibitor and BApNA substrate (0.625 and 1.25 mM). Figure 1C shows that JTI competitively inhibited trypsin. To determine the value of the inhibition constant (Ki), a Dixon plot was constructed [14]. The graph analysis confirms that JTI can inhibit trypsin in a competitive manner and with a Ki value of 1.7 × 10^−6^ M.

### 2.3 Determination of the Amino Acid Sequence of JTI

The identification of the primary sequence JTI was performed by MALDI‐ToF‐ToF mass spectrometry, where the peptide sequence comprising the inhibitor was analyzed (Figure S4). The primary sequence of the N‐terminal portion of JTI comprises two fragments after trypsinization. The first fragment comprises 11 amino acid residues, the second 12 residues, and the third 23 residues, providing a sequence coverage of 46 residues. When aligned with PjTI (a Kunitz‐type trypsin inhibitor from *Prosopis juliflora*) [15], this coverage indicates that JTI belongs to the Kunitz‐type inhibitor family (Table S1). The JTI 46 amino acid residues sequence possesses 59% of amino acid identity, 6% of amino acid showed strong similarity, and 6% of amino acid with weak similarity when aligned with PjTI.

### 2.4 Hemolytic Activity of JTI Against Human Erythrocytes

The toxicity of JTI against human erythrocytes was evaluated using 0.1, 0.5, 1.0, 1.5, 2.0, and 2.5 µM of the inhibitor. JTI showed no toxicity against human blood cells when measured in a colorimetric assay, evaluated in a spectrophotometer at 405 nm (data not shown).

### 2.5 Effect of JTI on Cell Viability and Proliferation

**FIGURE 2:**
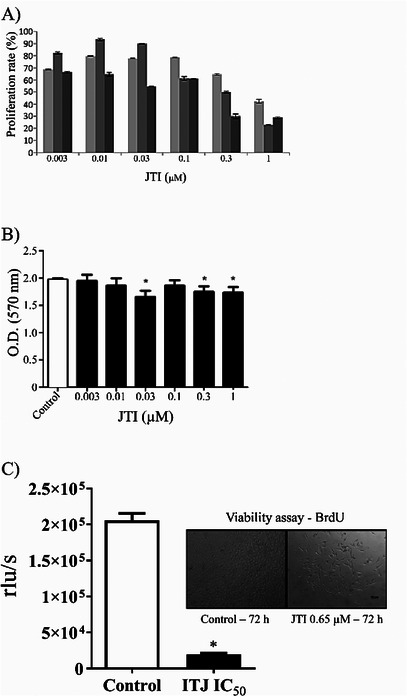
JTI in vitro cell culture assays: (A) JTI evaluation of cytotoxicity against B16‐F10 cells. Mouse melanoma cells (B16‐F10) were exposed for different times to JTI and evaluated by MTT assay. Gray scale from lightest to darkest represents 24, 48, and 72 h, respectively. (B) JTI cytotoxicity evaluation against a strain of murine fibroblasts. JTI tested at concentrations of from 0.003 to 1 µM; cells without the presence of the inhibitor were used as a negative control of the experiment. (*) *P* < 0.001. Values are expressed as the mean ± standard deviation (*n* = 3) compared to the control (no treatment). (C) BrdU incorporation into melanoma cells (B16‐F10) treated with JTI. Cells were treated for 72 h using the inhibitor IC_50_ for inhibition reviewed and analyzed by the BrdU incorporation method. (*) *P* < 0.001. Values are expressed as mean ± standard deviation (*n* = 3).

The activity of JTI on cell viability was investigated using MTT colorimetric assay [16]; Initially, HeLa (human cervix carcinoma), HepG2 (human hepatocellular carcinoma), and B16‐F10 (*Mus musculus* skin melanoma) tumoral cell lines were tested after incubation with increasing concentrations of JTI (0.003 to 1 µM), but only B16‐F10 tumoral cell line was affected after 72 h of exposure (Figure 2), with an IC_50_ of 0.65 µM.

The effect of JTI was also tested in a non‐carcinogenic fibroblastic cell line (3T3), but no toxicity was detected after 72 h of exposure (Figure 2). Due to the high incidence of melanoma in the population, JTI‐specific toxicity on this type of cell, and a significant reduction of cell proliferation, the B16‐F10 cell line was used for further tests. To verify a possible antiproliferative action resulting from incubation with the inhibitor, a proliferative marker, BrdU, was used (Figure 2) in mouse melanoma cells. JTI was able to inhibit tumor cell proliferation significantly in IC_50_ of 0.65 µM after 72 h incubation, not affecting the initial times of exposure.

### 2.6 JTI Induces Cell Death in Melanoma Cells by Incubation With Annexin‐V/ FITC and PI

Induction of apoptosis was visualized by flow cytometry, labeled with annexin V‐FITC/ PI. After analyzing the flow cytometry data, it could be seen that there is no significant difference in control cells (without contact with JTI) concerning cells that were exposed to JTI action in times of 24 and 48 h, but after 72 h the inhibitor showed strong indication to induce cell death in B16‐F10 cells, with positive double staining for annexin V‐FITC/ PI of approximately 34%.

**FIGURE 3:**
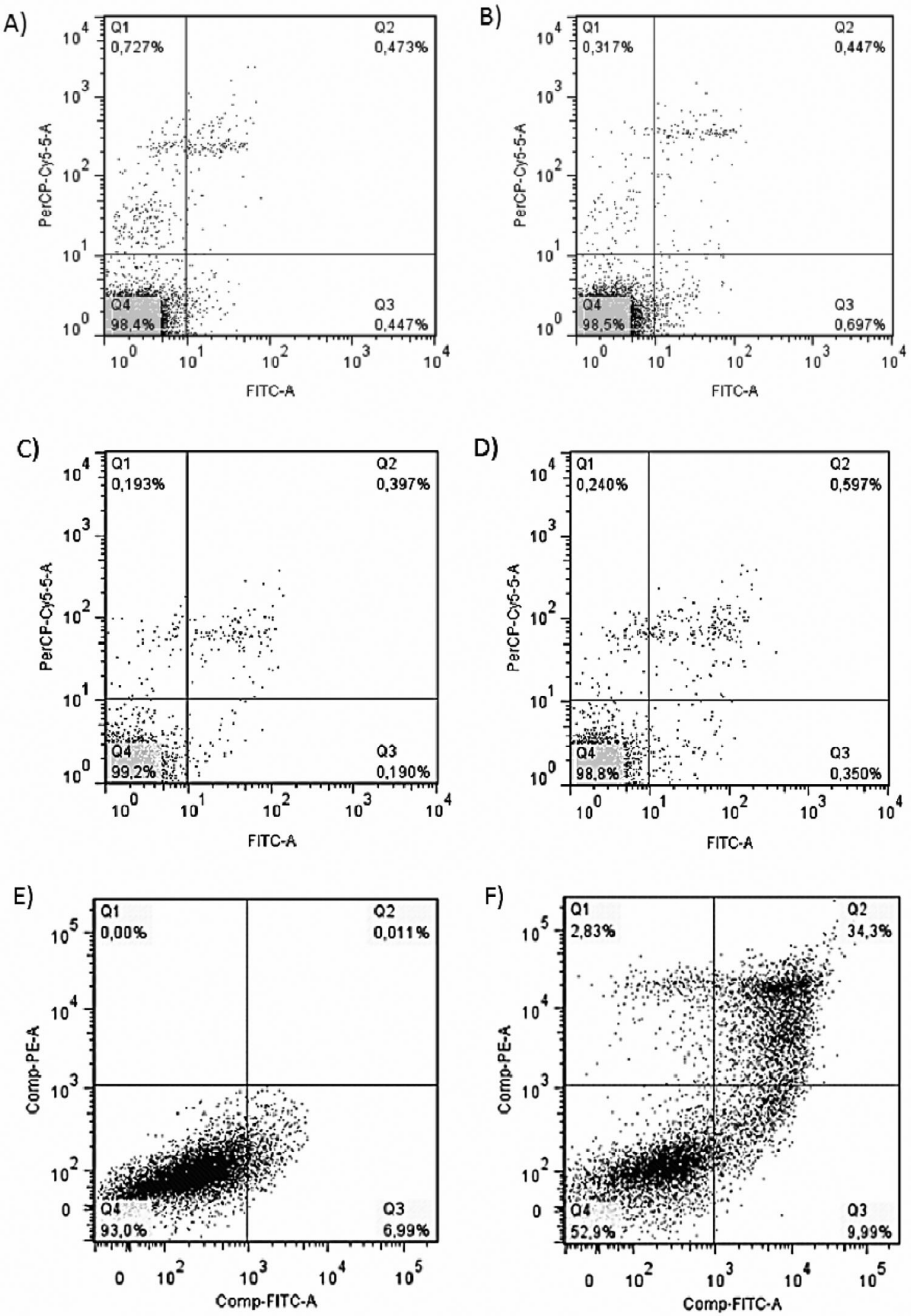
Annexin V and propidium iodide under JTI action on melanoma cells. (A) Control cells 24 h; (B) cells treated with 0.65 µM of JTI for 24 h; (C) cells control 48 h; (D) cells treated with 0.65 µM of JTI for 48 h; (E) cells control 72 h; (F) cells treated with 0.65 µM of JTI for 72 h. FL2H‐ filter relating to propidium iodide. FL1H‐ filter relating to annexin V‐FITC. The results represent the average of three separate experiments.

It was observed that cells were present in both the initial stage of apoptosis (positive staining for annexin V‐FITC) and in the late apoptosis stage (positive for propidium iodide). Approximately 10% of tumor cells exposed to JTI were positively stained for annexin V‐FITC, indicating that they were progressing to the next stages of apoptosis. Comparing the control cells with those exposed to the inhibitor, nearly 50% of the cells showed activation of the cell death mechanism through apoptosis (Figure 3).

### 2.7 JTI Effects on the Cell Cycle Course

After incubation for 72 h with JTI, using a fixed concentration of 0.65 µM, there was an accumulation of arrested cells in the G1 phase of the cell cycle (85.41%), which indicates the characteristic direction of tumor cells to initiate the process of cell death by apoptosis (Table 2).

**TABLE 2:** Cell cycle arrest evaluation of melanoma cells after exposure to JTI at different times.

Phases	Control	JTI	Control	JTI	Control	JTI
	24 h	48 h	72 h	24 h	48 h	72 h
G1	59.15	68.84	60.37	66.1	68.77	85.41
S	29.89	24.81	10.51	17.68	20.21	11.97
G2	3.38	2.41	1.85	7.35	2.23	2.62
Sub‐G1	5.43	5.3	22.35	10.15	8.53	1.14
Super‐G2	1.25	1.01	2.11	1.26	0.47	1.93
RMS	9.38	10.3	9.45	17.39	17.79	13.16

After 72 h of treatment with JTI, B16‐F10 cells were fixed, treated with RNAse, and stained with propidium iodide and analyzed by flow cytometry to evaluate cell cycle distribution. Data analysis for cell cycle arrest was performed using FlowJo software v. 7.6.3.

The Western blot technique was used to analyze the expression of proteins related to the cell cycle (expression of cyclins B1, D1, D3, E, and p21) under JTI action. JTI, a fixed concentration (0.65 µM), was incubated with B16‐F10 cells (Figure S5) at different exposure times (control, 24, 36, 48, 60, and 72 h). After incubation with JTI, cyclins D1, D3, and E were downregulated, but strangely, cyclin B1 had to be increased, especially in the last measured time.

The reduction of the expression of cyclins D and E caused by the inhibitor should induce the stop of the melanoma cells in the G1 phase of the cell cycle, apoptosis, confirming the theory suggested in this work. In addition to significantly altering the expression of cyclins, JTI was also effective in increasing the expression of the proteins p21 and p53, related to the inhibition of cell proliferation in response to DNA damage.

The p53, when activated, induces the expression of the p21 protein, which binds to and inhibits the dependent kinase activity of cyclin, preventing the phosphorylation of critical cyclin‐dependent kinase substrates, and blocking cell cycle progression (Figure S6). The high expression inhibits the p21 kinase activity of the CDK4‐cyclin D complex, confirming the results obtained in the Western blot (Figure S5).

### 2.8 Evaluation of Mitochondrial Membrane Potential Change (ΔΨm) Induced by JTI

The JTI effect on the change in mitochondrial membrane potential was evaluated by flow cytometric technique (using the probe Rhodamine 123) and by confocal microscopy (using Mito Tracker Red probe). The first assay was performed using flow cytometric techniques using the Rhodamine 123 probe. B16‐F10 cells were divided into two groups: one group underwent treatment with JTI at 0.65 µM, and one without treatment, which was used as a negative control.

After incubation with Rhodamine 123, it was observed that JTI was able to significantly reduce the mitochondrial electrical potential, even after 72 h of treatment, revealing the toxicity of the inhibitor to such cells (Figure S7). The second methodology was used to probe Mito Tracker Red as a potential change indicator of mitochondrial membrane, visualized in a confocal microscope, Leica SP8.

**FIGURE 4:**
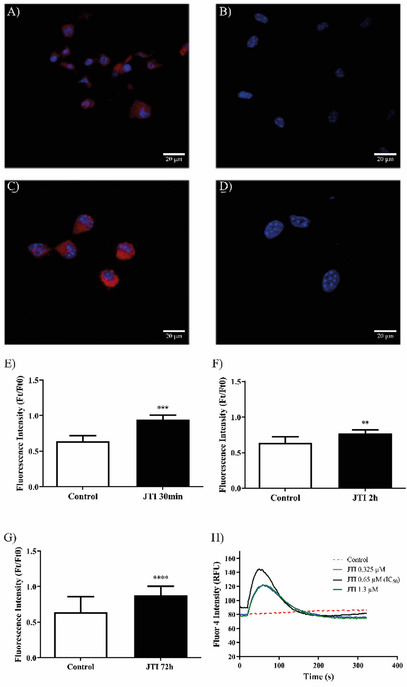
JTI effect on the mitochondrial membrane potential, ROS release, and cytosolic levels of Ca^2+^ in B16‐F10 cells. (A–D) Confocal microscopic analysis on the mitochondrial potential, A and C: untreated cells (control); B and D: cells treated for 72 h with 0.65 µM JTI; C and D: Zoom 1.5x; red—mitochondria treated with Mito Tracker Red; blue—nuclei stained with DAPI. (E–G) JTI effect on the release of ROS by melanoma cells. The release of ROS by B16‐F10 cells under action JTI was determined by the H_2_DCFDA method. (E) Cells treated for 30 min with JTI; (F) cells treated for 2 h with JTI; (G) cells treated with JTI for 72 h of exposure. **** *P* ≤ 0.0001; *** *P* ≤ 0.001; ** *P* ≤ 0.01. (H) JTI influence on the levels of cytosolic Ca^2+^. Assay performed using the Fluo‐4 Calcium Assay Direct Kit (F10471, Invitrogen, UK). *P* < 0.0001.

B16‐F10 cells were incubated with Mito Tracker Red probe following treatment (or not) with JTI (the concentration IC_50_) and analyzed by confocal microscopy. The analysis showed that the mitochondrial membrane potential of tumor cells was lost after 72 h of inhibitor exposure (Figure 4).

### 2.9 JTI Influences the Release of Reactive Oxygen Species (ROS) and Nitrogen (RNSs)

Intracellular ROS production by the protease inhibitor was evaluated by the dichlorofluorescein acetate method (H2DCFDA), which is converted into reduced dichlorofluorescein (DCFH) in the cytoplasm and is oxidized by ROS to dichlorofluorescein (DCF), which emits fluorescence. JTI showed a potent prooxidant effect on B16‐F10 cells after 30 min, 2 h, and 72 h of exposure to 0.65 µM (Figure 4), being a more effective effect than the first time reported. JTI was not able to alter the expression of RNSs at any time evaluated, as observed after incubation with a DAF‐FM probe (difluorofluorescein) (data not shown).

### 2.10 JTI Influences the Increase in Calcium Levels in Cells B6‐F10

The cells were incubated with Fluo‐4 Direct reagent for 1 h at 37°C for incorporation, and then fluorescence reading was performed in a microplate reader every 2 s for at least 300 s. An injection of the sample was made for it to normalize its optimal concentration in the time of 20 s, where it was observed that, at 0.65 µM, there was a more significant increase in cytosolic Ca^2+^ (Figure 4), which is possibly related to the initiation of cell death process of B16‐F10 cells via signaling mechanisms, along with the release of ROS. After 72 h of exposure to the inhibitor, there was no change in the levels of cytosolic Ca^2+^.

### 2.11 JTI Influences the Expression of Proteins Related to Apoptosis in B16‐F10 Cells

**FIGURE 5:**
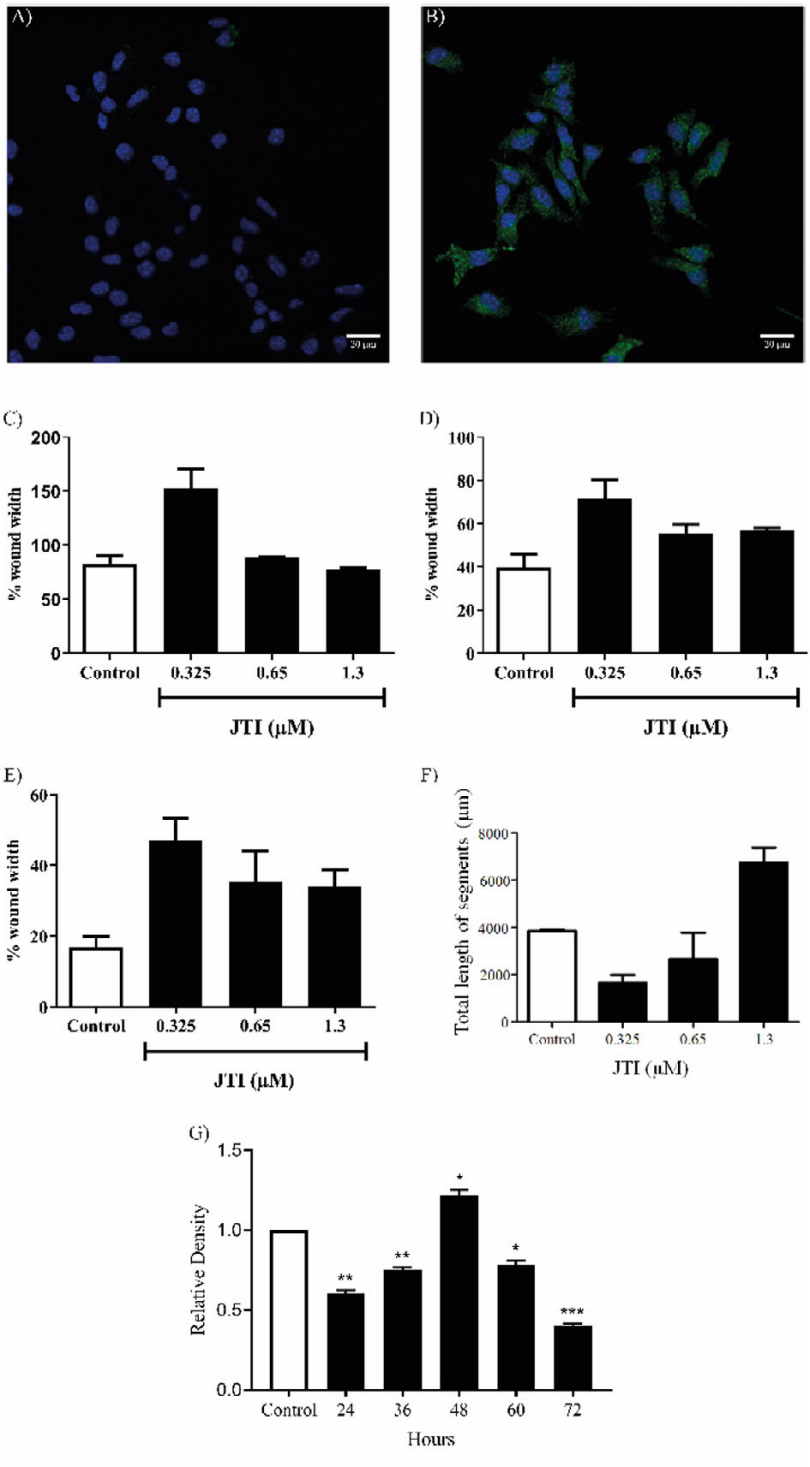
JTI induces apoptosis and inhibits migration and angiogenesis in melanoma cells. (A,B) Caspase 3 expression in B16‐F10 cells after treatment with JTI. Cells were plated onto coverslips (13 mm diameter) and cultured for three days as described in Methods, and subsequently incubated in the presence or absence of JTI. Caspase‐3 staining was obtained using cleaved caspase‐3 antibody (Asp175) (5A1E) (# 9664) Rabbit mAb. The images were captured on a Leica confocal microscope SP8. Nuclei stained with DAPI (blue) and caspase‐3 (green). Representative images of three separate assays. A—control; B—treatment with JTI (0.65 µM) in 72 h of exposure. (C,D) JTI effect on cell migration of B16‐F10. A monolayer culture of B16‐F10 was established, where an injury was made and subsequently treated with JTI at a concentration of 0.325, 0.65, and 1.3 µM in various ranges of exposure times, (C) 3, (D) 6, and (E) 18 h. The injury was measured and compared between the various treatments and exposure times to the inhibitor. (F,G) JTI effect on angiogenesis. (F) Endothelial cells (RAEC) were cultured on Matrigel polymerized in the presence of F12 medium containing 10% FBS for 72 h in the presence of different concentrations of JTI. The formation of the tubes was examined in an inverted light microscope using an objective 5X. (G) Inhibition of VEGF expression after 72 h of incubation with the concentration IC_50_ of JTI; trial using the Western blotting technique, where the analysis of the protein was normalized by the β‐tubulin in B16‐F10 cells. Densitometry of the expressed protein was performed using ImageJ software. **** P ≤ 0.0001; *** *P* ≤ 0.001; ** *P* ≤ 0.01, * *P* ≤ 0.05.

After exposure to the trypsin inhibitor, p53 was stimulated, inducing B16‐F10 cells to stop proliferating and begin the process of cell death by apoptosis. However, after extended exposure times, its expression showed a slight decrease (Figure S8). In addition to altering the expression of p53 in tumor cells, JTI reduced the expression of Erk, influencing the induction of B16‐F10 cell death after 72 h of exposure. The JTI protease inhibitor was also able to induce significant activation of effector caspase 3 after 72 h of apoptotic exposure (Figure 5A,B).

### 2.12 JTI Effects on the Migration of B16‐F10 Cells

The migration percentage of B16‐F10 cells was assessed with different concentrations of JTI (0.325, 0.65, and 1.3 µM) at various exposure times (0, 3, 6, and 18 h). Both IC_50_ and twice the IC_50_ showed a significant effect on cell migration processes compared to their respective controls, although they were lower than the result obtained with 0.325 µM of JTI (Figure 5C,D).

### 2.13 JTI Effects on Angiogenesis and Analysis of VEGF and IL‐6 Expression in B16‐F10

The influence of JTI on the formation of new vessels in the Raec cell line was analyzed. It was shown that JTI inhibits angiogenesis at concentrations equivalent to the IC_50_ (0.65 µM) and half of the IC_50_ (0.325 µM). However, when a concentration equivalent to twice the IC_50_ was applied, a stimulatory effect on new vessel formation was observed in the test cells, indicating that the observed inhibitory effect of angiogenesis by the inhibitor is dose‐dependent (Figure 5F).

VEGF is a growth factor involved in angiogenesis, vasculogenesis, and endothelial cell growth. It induces endothelial cell proliferation, promotes cell migration, inhibits apoptosis, and increases the permeability of blood vessels. After 72 h of exposure to the inhibitor (0.65 µM), a decrease in VEGF expression was observed, which, together with the inhibition assay of capillary‐like structure formation in matrigel, indicates a reduction in the angiogenic process in melanoma cells (Figure 5G).

**FIGURE 6:**
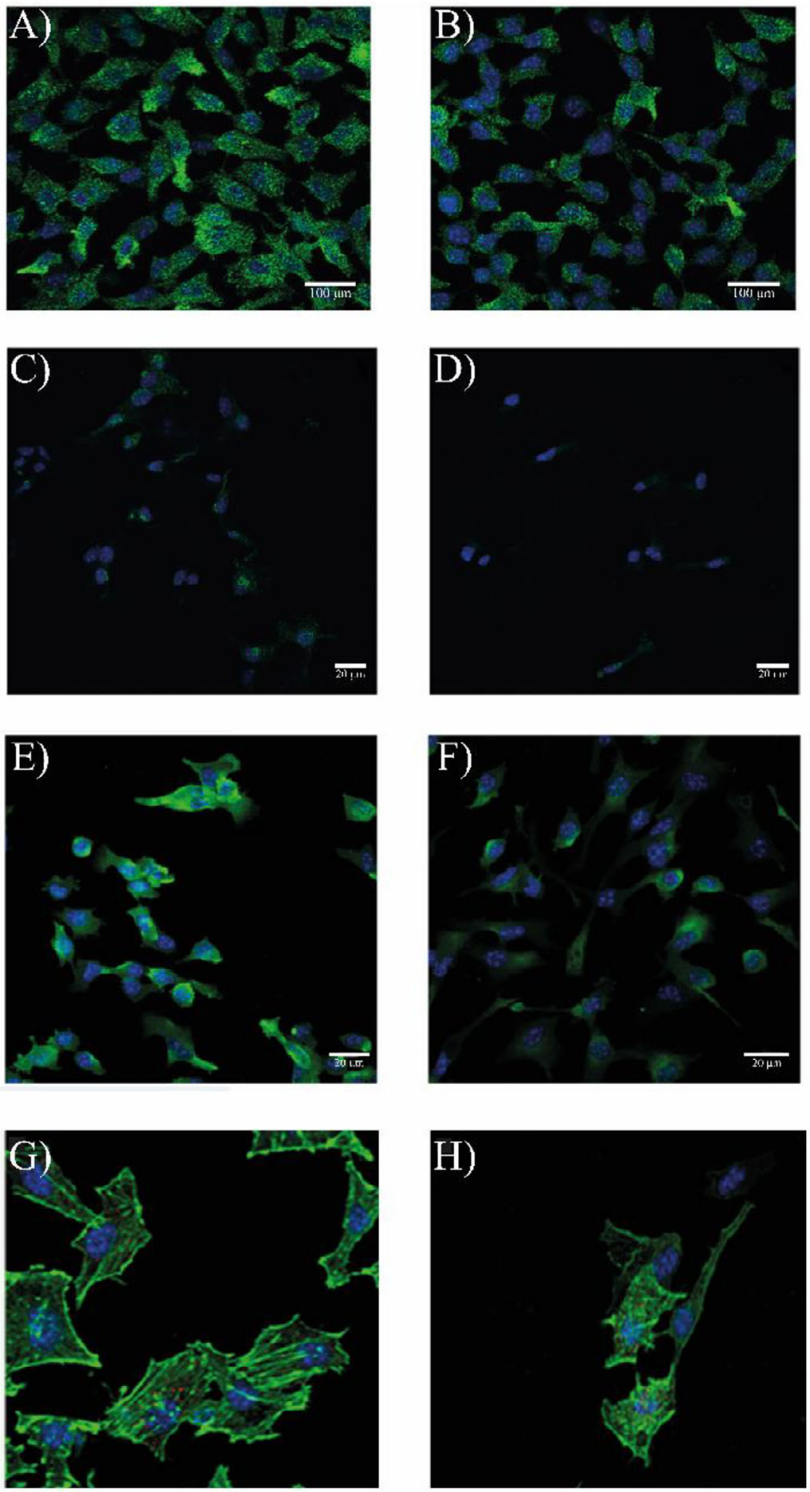
JTI alters inflammatory signaling and cytoskeletal organization in B16‐F10 melanoma cells. (A,B) JTI effect on IL‐6 expression in B16‐F10 cells. (A) control; (B) treatment with JTI (0.65 µM) in 72 h of exposure; cells were plated onto coverslips (13 mm diameter) and cultured for three days as described in Methods, and subsequently incubated in the presence or absence of JTI. The marking of interleukin‐6 was obtained using IL‐6 antibody (SC‐130326). Nuclei stained with DAPI (blue) and IL‐6 (green). (C,D) Vimentin expression in B16‐F10 cells exposed to JTI. The marking of the vimentin intermediate filament was obtained using vimentin antibody (sc‐73259) in conjunction with Alexafluor 594. Nuclei stained with DAPI (blue) and vimentin (green). (C) control; (D) treatment with JTI (0.65 µM) in 72 h of exposure. (E,F) JTI effect on the expression of chondroitin sulfate in JTI B16‐F10 cells. After 72 h of treatment, the pattern of organization of intermediate filaments in melanoma cells is modified by changing the cell morphology. (E) Control; (F) treatment with JTI (0.65 µM) in 72 h of exposure; cells were plated onto coverslips (13 mm diameter) and cultured for three days as described in Methods, and subsequently incubated in the presence or absence of JTI. The marking of the intermediate filament chondroitin sulfate was obtained using chondroitin antibody (370,615) combined with Alexafluor 594. Nuclei stained with DAPI (blue) and chondroitin sulfate (green). Images represented for two overlayer assays. **G‐H)** Phalloidin and COX IV expression pattern in B16‐F10 cells after treatment with JTI. (G) control; (H) treatment with JTI (0.65 µM) in 72 h of exposure; Cells were plated onto coverslips (13 mm diameter) and cultured for three days as described in Methods, and subsequently incubated in the presence or absence of JTI. The markings of phalloidin and COX IV were obtained using the Phalloidin antibodies conjugated with Alexa Fluor 488 (A12379) and COX‐IV (48505). Nuclei stained with DAPI (blue), phalloidin (green), and COX IV (red). All images were captured on a Leica confocal microscope SP8, and representative images of three separate assays.

Treatment with JTI significantly reduced IL‐6 expression in melanoma cells after 72 h of treatment (Figure 6A,B). This result, along with the reduction in capillary formation in matrigel, may indicate the involvement of JTI in inhibiting the angiogenic process, either directly or indirectly, by reducing the expression of the pro‐inflammatory cytokine IL‐6.

### 2.14 JTI Alters the Expression of the Intermediate Filaments and ECM Components in Melanoma Cells

After 72 h of exposure, the control (Figure 6C) was compared to the treatment assay, where JTI at 0.65 µM was evaluated for vimentin expression (Figure 6D), suggesting a significant role as an inhibitor of melanoma progression in B16‐F10 cells. Chondroitin sulfate is a glycosaminoglycan that is synthesized and comprises the side chain of proteoglycans. There is substantial evidence suggesting the involvement of chondroitin sulfate, as well as heparan sulfate, in the development of the central nervous system, wound repair, infectious processes, growth factor signaling, morphogenesis, and cell division [17].

After 72 h of exposure, the control (Figure 6E) was compared to the treatment assay, revealing a reduction in chondroitin sulfate expression in melanoma cells (Figure 6F), along with a visible change in its distribution pattern. Initially, expression appeared more rounded, but following treatment with the inhibitor, the cells exhibited an elongated, needle‐like morphology.

The change in the intensity of β‐actin expression, along with the analysis of the morphological changes in the arrangement of actin in melanoma cells (Figure 6G,H), corroborates the results obtained to assess the influence of the protease inhibitor on the cell migration process. When there is a change in the actin expression pattern, likely, there is also a change in the cell migration pattern. Cytochrome c oxidase subunit IV (COX IV) catalyzes the final stage of the mitochondrial electron transfer process and is considered one of the most important regulatory sites of oxidative phosphorylation.

Interestingly, suppression of COX IV expression also sensitizes cells to apoptosis. We observed a slight decrease in COX IV expression, which confirms the data obtained from the change in mitochondrial potential (Figure 6G,H). This suggests that B16‐F10 cells are being induced to cell death due to the action of JTI.

## 3 Discussion

In recent decades, a large number of studies have unraveled the signaling pathways involved in the control of cell death and the molecular machinery responsible for this process, leading to numerous possibilities for pharmacological intervention and drug design [18]. Among the new drugs used in cancer therapy, natural compounds such as carbohydrates, phenolics, antimicrobial peptides, lectins, enzyme activity regulators, and peptidase inhibitors have gained attention as important sources of chemotherapeutic agents and/or as chemical and structural models for the development of various compounds [19].

The present study investigated the effects of JTI, a purified trypsin inhibitor from the seeds of *M. regnellii*, on mouse melanoma cells. Purification was carried out from total seed extracts through ammonium sulfate precipitation, followed by affinity chromatography, ion exchange, and RP‐HPLC, as these techniques have been used in the purification of other plant‐derived protease inhibitors [4].

Although the N‐terminal sequence coverage obtained by MALDI‐ToF/ToF is partial (∼25%), the high homology (up to 83%) with established Kunitz‐type inhibitors and the identification of the conserved Cys^38^ residue provide compelling evidence for JTI's classification. When integrated with its functional inhibitory profile and the molecular weight (consistent with the ∼20 kDa range), these biochemical hallmarks robustly identify JTI as a Kunitz‐type protease inhibitor. Consequently, this characterization provides a solid foundation for investigating its biological effects and therapeutic potential in melanoma cells [4, 15, 20].

The identified N‐terminal region shares high homology with well‐documented Kunitz‐type inhibitors [15]. More specifically, 79% with PjTI from *Prosopis juliflora* [15], 83% with *Senna tora* Kunitz‐type trypsin inhibitor alpha chain‐like, 81% with DE5 alpha chain‐like from *Neltuma alba*, 81% EcTI from *Enterolobium contortisiliquum*. Furthermore, multiple sequence alignment revealed the presence of a conserved cysteine residue (Cys^38^), a structural hallmark of the Kunitz‐type family, further reinforcing this taxonomic assignment [20].

However, the absence of a full‐length sequence represents a limitation in defining cysteine disulfide bridges and secondary structure; therefore, JTI definition as a Kunitz‐type inhibitor is based on its convergence of biochemical activity, partial sequence attributes, and characteristic molecular weight [4, 6]. SDS‐Page analysis under reducing conditions with DTT showed a shift in the JTI migration pattern (Figure S2), which can indicate the presence of a disulfide bridge [21]. Further studies with primary sequence data obtained through transcriptomic or deeper proteomics analysis are required to provide definitive sequence, structure, and disulfide bond connectivity.

Furthermore, JTI demonstrated superior potency against tumor cells when compared to other plant‐derived inhibitors. For instance, the purified trypsin inhibitor from *Nephelium lappaceum* seeds [22] exhibited toxicity against various tumor cell lines with IC_50_ values of 130.7 µM (MCF‐7), 215.3 µM (HepG2), 277 µM (CNE‐1), and 30 µM (CNE‐2). Similarly, a purified trypsin inhibitor from *Phaseolus vulgaris* [23] showed toxicity against MCF‐7 and HepG2 cells at an IC_50_ of 125 µM. By comparison, JTI is 46‐ to 426‐fold more potent than *N. lappaceum* and 192‐fold more potent than *P. vulgaris* inhibitor. BrdU assay further confirm JTI efficacy at inhibiting tumor cell proliferation, this contrasts with other inhibitors like ECTI, which significantly reduced cancerous gastric cells (Hs746T) viability by 37% (up to 150 µM) but didn't affect cellular proliferation after 24 and 48 h of incubation [24].

Despite the antitumor activity of JTI, it showed no toxicity when incubated with erythrocytes, fibroblasts (3T3), and endothelial cells (Raec). This safety profile in the tested concentrations is consistent with other characterized trypsin inhibitors, such as those from *Coccinia grandis* [25], *Erythrina velutina* (EvTI), *Xanthosoma blandum* (xb‐KTI) [26], and *Enterolobium contortisiliquum* [24]. Similar nontoxic results were reported for the PIVL inhibitor [27] and the BMTI‐A inhibitor from *Rhipicephalus microplus* [28], which caused no cytotoxicity in HMEC‐1 and HUVEC cells, respectively, after 72 h of incubation (at 0.1 to 1 µM).

A screening was carried out to determine the potential activities of the purified material in modifying cellular events. Assays with tumor cell lines HeLa, HepG2, and B16‐F10, as well as normal 3T3 and Raec cells, were performed to evaluate the toxicity induced by incubation with JTI. It was observed that the inhibitor exhibited significantly higher toxicity compared to other purified inhibitors from leguminous seeds, such as the chymotrypsin inhibitors from *Acacia confusa* [29] and purified trypsin *Vigna unguiculata* seeds, called BTCI [30], which were cytotoxic against MCF‐7 tumor cells with an IC_50_ of 10.7 and 200 µM, respectively. When quantitatively comparing the IC_50_, JTI presents an antitumoral IC_50_ 16‐fold and 300‐fold more potent than *A. confusa* and *V. unguiculata* inhibitors, respectively. Furthermore, similar effects on cell viability, proliferation, and the cell cycle were found in other Fabaceae‐derived Kunitz seed inhibitors [4, 31, 32].

Apoptosis can be triggered by various intracellular stress conditions and morphological changes, including DNA damage, oxidative stress due to an overload of cytosolic Ca^2+^, excitotoxicity, the accumulation of unfolded proteins in the endoplasmic reticulum (ER), and many others. BPLTI, a purified inhibitor from *Bauhinia purpurea* L. seeds, was capable of inducing apoptotic cell death in HepG2 liver cells [33]. When incubated at concentrations ranging from 0 to 150 µM, VFTI‐G1, an isolated inhibitor from *Vicia faba*, was also able to induce apoptotic cell death in the HepG2 tumor cell line at several doses ranging from 7.5 to 90 µM after 24 h of exposure. Additionally, the BCTI inhibitor induced cell death, marked by annexin V‐FITC, after 72 h of incubation with 200 µM in MCF‐7 cells [30], but JTI proved far more effective in inducing apoptosis at 0.65 µM tested.

The varying response times of inhibitors on tumor cells can be attributed to possible differences in their mechanisms of action, which may explain the rapid effect of some inhibitors compared to the delayed effect of JTI in inducing cell death by apoptosis in B16‐F10 cells. After incubation with JTI, cyclins D1, D3, and E were downregulated, while surprisingly, cyclin B1 was upregulated, particularly at the later time points. In addition to significantly altering the expression of cyclins, JTI was also effective in increasing the expression of the proteins p21 and p53, which are associated with the inhibition of cell proliferation in response to DNA damage.

Activation of p53 at 36 h induces the expression of p21, which binds to and inhibits cyclin‐dependent kinase (CDK) activity, preventing phosphorylation by CDKs and thereby blocking cell cycle progression. Despite the increase in p53 expression at 36 h, the protein is downregulated at later time points, suggesting that while p53 may have been involved in the activation of p21, it was not crucial for its sustained expression. Due to its high expression, p21 inhibits the kinase activity of the CDK4‐cyclin D complex, which supports the results obtained from Western blot analysis (Figure S5).

JTI demonstrated the ability to induce a loss of mitochondrial membrane potential (ΔΨm), which was accompanied by a high number of apoptotic cells, highlighting the importance of mitochondrial depolarization by JTI in mediating apoptosis. In comparison to JTI treatment, 200 µM of BCTI was used in the evaluation of mitochondrial membrane potential in MCF‐7 cells [30], and GBP‐TI, which was capable of causing changes in mitochondrial membrane potential after 24 h of exposure to 20 µM of the inhibitor, was much less effective than that found for JTI, affirming the high toxicity and effectiveness of this inhibitor when administered at low dosages (0.65 µM), changes in the mitochondrial membrane potential (ΔΨm) of mouse melanoma cells [34].

JTI exhibited a potent pro‐oxidant effect on B16‐F10 cells. When compared to RBTI, JTI was significantly more effective in inducing oxidative stress through the release of reactive oxygen species (ROS). Another compound known to induce ROS production is staurosporine [35], which was evaluated using the same method as JTI. However, JTI proved to be more effective in generating ROS than staurosporine. At a concentration of 0.65 µM, JTI caused a substantial increase in cytosolic Ca^2+^ levels, potentially initiating the cell death process in B16‐F10 cells via signaling mechanisms, in conjunction with ROS release. Other protease inhibitors, such as BBCI [36], also influenced cytosolic calcium levels, but were much less effective than JTI.

Regarding the angiogenesis assays, JTI has shown a complex, biphasic dose‐dependent profile. In lower concentrations (≤ IC_50_ = 0.65 µM), it effectively inhibited tube formation; however, in supra‐pharmacological concentrations (2 x IC_50_ = 1.3 µM), it appears to promote transient cellular extensions. This dual effect may be mechanistically associated with the rapid pro‐oxidant activity and the increase of cytosolic Ca^2+^ levels triggered by JTI exposure. The massive influx of calcium, an important second messenger in endothelial cell mobility and cytoskeleton remodeling [37] may paradoxically activate short‐term compensatory migratory signaling and survival pathways before the onset of apoptosis [38]. This phenomenon, often described as hormesis [38], suggests that the antiangiogenic potential of JTI can be optimized within a specific therapeutic window, beyond which can lead to off‐target effects and the disruption of cellular homeostasis, which can lead to nonlinear biological responses [39].

Similar results to those achieved with the *M. regnellii* trypsin inhibitor were obtained by incubating Amblyomin‐X (0.5 µM) for 5 and 24 h in RENCA cells [40]. Preincubation with 0.65 µM of JTI was much more effective in slowing down cell migration and angiogenesis compared to other protease inhibitors, such as ECTI (*Enterolobium contortisiliquum* trypsin inhibitor), which at 100 µM reduced cell migration by approximately 30% in Hs746T gastric cancer cells [24]; TFPIc23 was less effective in reducing the angiogenic process compared to JTI, highlighting the potency of this inhibitor in limiting the formation of blood vessels necessary for tumor nutrition. Vixapatin (VP12), a C‐type lectin purified from the venom of Vipera xanthine palestinae [41] yielded results similar to JTI, but at a slightly higher dose (1 µM).

While several Kunitz‐type protease inhibitors from the Fabaceae family have been characterized for their anticancer potential [4, 6], the JTI represents and novel addition to the Fabaceae‐derived protease inhibitors, due to its origin from a non‐characterized plant in endemic South American regions [42] and its biological activity in a highly metastatic, adherent, murine melanoma cell line B16‐F10 [43, 44] and other non‐cancerous cell lines.

JTI stands out due to its remarkable submicromolar efficacy, targeting B16‐F10 melanoma cells while sparing fibroblasts and endothelial cells. Distinctively, JTI exerts a multitargeted effect by downregulating VEGF and IL‐6, and triggering early ROS and Ca^2+^ fluxes as primary signals that precede mitochondrial dysfunction and G1‐phase arrest [37, 45]. Furthermore, its ability to disrupt vimentin and chondroitin sulfate organization provides a comprehensive correlative mechanistic perspective that is infrequently reported for other family members. These findings clearly distinguish JTI from previously described inhibitors.

## 4 Conclusion

In conclusion, the inhibitory effect of JTI on melanoma cell migration was demonstrated in comparison with known protein inhibitors associated with angiogenesis, cell differentiation, and migration. Our findings provide initial evidence supporting the antitumoral potential of JTI and suggest its involvement in apoptotic‐related pathways leading to melanoma cell death. Although our data suggest the involvement of mitochondrial‐associated apoptotic pathways, the current evidence remains correlative. Additionally, a dual effect on angiogenesis was observed for JTI. This behavior provides a therapeutic window to exert both pro‐ and anti‐angiogenic properties at supra‐pharmacological and IC_50_ concentrations, respectively.

However, we acknowledge that the present study represents an initial step toward correlative elucidating its mechanism of action. Additional investigations, including more caspase inhibition assays, rescue experiments using ROS and RNS scavengers or Ca^2+^ chelators, and direct assessment of cytochrome c release, will be essential to more precisely define the molecular pathways involved. These complementary approaches are of great importance and may be addressed in future studies through collaboration with specialized research groups.

## 5 Experimental Section/Methods

### 5.1 Biological Material

The seeds of Juquiri legume (*Mimosa regnellii* Benth) were provided by the Division seed bank, Technical National Nísia Floresta, Chico Mendes Institute for Biodiversity Conservation—ICMBio / MMA. Human erythrocytes were obtained from blood bags of donations by HEMOCENTRO—RN. The supplied bags found themselves outdated for transfusions. B16‐F10, a cell line of mouse melanoma (ATCC CRL‐6475 number), and 3T3, a normal mouse fibroblast line (ATCC CL‐173 number), were obtained from American Type Culture Collection (ATCC, Rockville, MD, USA). Raec cell line, composed of endothelial cells from the rabbit aorta, was kindly provided by Prof. Dr. Helena Bonciani Nader, UNIFESP / SP. The B16‐F10 and 3T3 lines were cultured in DMEM (Dulbecco's Modified Eagle's Medium) supplemented with 10% fetal bovine serum, streptomycin (5000 U.mL^−1^):penicillin (5000 IU). The cells were maintained in a sterile environment with 5% CO_2_, while the Raec cell line was cultured in F12 medium GIBCO (Grand Island, NY): A nutrient mixture containing 2 mM L‐glutamine, sodium bicarbonate 3.7 g L^−1^, penicillin 10,000 U.L^−1^ streptomycin, and 10 mg L^−1^ (Sigma Chemical CO., St. Louis, MO, USA). The F12 culture medium was supplemented with 10% fetal bovine serum (FBS) (Cultilab, Campinas, São Paulo, Brazil). The cells were kept under sterile conditions with 2.5% CO_2_.

### 5.2 Inhibitor Purification

Seeds were peeled to obtain fine‐grained flour, homogenized in Tris‐HCl 0.05 M buffer, pH 7.5, 1:10 (w:v) under constant stirring for 3 h at room temperature. The homogenate was centrifuged at 8,000 x g for 30 min at 4°C, resulting in a supernatant called Crude Extract (CE). CE was fractionated in three ammonium sulfate concentration ranges: 0%–30% (F1), 30%–60% (F2), 60%–90% (F3), and left overnight; it was then centrifuged at 8000 x g for 30 min. The obtained precipitates were dissolved in tris‐HCl 0.05 M buffer, pH 7.5. All fractions were tested for the detection of inhibitory activity against serine proteases, and F2 showed the highest inhibitory activity, being chosen for further steps of purification. This soluble fraction was applied to a CNBr‐Sepharose 4B trypsin affinity column, pre‐equilibrated with 0.02 M sodium tetraborate buffer, pH 7.5. After extensive washing with the equilibration buffer, the inhibitor was eluted with 0.1 M HCl, and the inhibitor activity was measured by the inhibition of trypsin hydrolysis of 1 mM BApNA (Benzoyl‐DL‐arginyl‐*p*‐nitroanilide) (Bachem, Bubendorf, Switzerland) as substrate [46, 47]. The fractions containing the inhibitory activity were pooled and applied sequentially to a DEAE‐Sephadex column equilibrated with 0.02 M sodium tetraborate buffer, pH 7.5, and the retained proteins were eluted with 0.3 M NaCl. The last step of the purification was a reverse phase chromatography in a C8 column developed with an acetonitrile gradient (0%–100%) in trifluoroacetic acid (TFA) (0.1%, v:v) at room temperature at a flow rate of 0.7 mL min^−1^; the isolated peak was named JTI. The homogeneity and the molecular weight of the inhibitor were assessed by 15% SDS‐polyacrylamide gel electrophoresis [48].

### 5.3 Protein Quantification

Proteins were quantified by the BCA method (bicinchoninic acid, Thermo Scientific) [49], using bovine serum albumin (BSA) as a standard. The absorbance readings are performed at 562 nm in a spectrophotometer Pharmacia Biotec (mod, Ultrospec 2100 – pro).

### 5.4 Anti‐Trypsin activity

The anti‐trypsin activity of JTI from fractions to the final purification steps was determined using BApNA as a substrate [46, 47]. Aliquots of 10 µL bovine trypsin solution (0.3 mg mL^−1^ in tris‐HCl 0.05 M, pH 7.5 buffer) were preincubated for 10 min at 37°C with 120 µL HCl 0.0025 M, 270 µL tris‐HCl 0.05 M buffer, pH 7.5, and 100 µL inhibitor. After this time, the reaction was started by adding 500 µL BApNA. The reaction continued for another 15 min under the same incubation conditions and stopped by adding 120 µL of 30% acetic acid. The formation of p‐nitroaniline was monitored in a spectrophotometer at 410 nm. Blank tests were carried out, and the tests were performed in triplicate and repeated three times. The results were expressed as IU mg^−1^ protein.

### 5.5 IC_50_ and Ki Determination Against Bovine Trypsin

Increasing concentrations of JTI were incubated with 20 µL trypsin, and an enzyme assay was performed as previously described [46, 47]. The percentage of trypsin inhibitor for each inhibitor concentration was used to construct a titration curve and determine the IC_50_ (half‐maximal inhibitory concentration). To determine the mode of inhibition and inhibition constant (Ki), data were plotted according to Dixon [14] as the intercepts between two plot lines using two BApNA in 0.625 and 1.25 mM. The enzymatic reaction velocity (V) was expressed as reaction product optical density (OD) at 405 nm as a function of reaction time (h) and volume (mL) (*V*   =   OD h^−1^ mL^−1^). Assays were performed as previously described.

### 5.6 Structural Stability in the Presence of Denaturants and Reducing Agents

The evaluation of the stability of JTI in the presence of SDS and DTT (dithiothreitol), followed by the methodology described by Mello [50] with modifications. Samples of the inhibitor were incubated in a water bath separately, with 10% SDS and 25 mM DTT for 30 min at 100°C. After cooling the samples to 4°C, a polyacrylamide gel electrophoresis on 15% was performed to pattern analysis of protein banding analysis.

### 5.7 Amino Acid Sequence Determination

JTI molecular mass was determined by using MALDI‐ToF‐ToF analysis (UltraFlex III, Bruker Daltonics, Billerica, MA). Purified JTI was dissolved in a minimum volume of water that was mixed with an α‐cyano‐4‐hydroxycinnamic acid saturated matrix solution (1:3, v:v), spotted onto a massive target plate, and dried at room temperature for 5 min. The α‐cyano‐4‐hydroxycinnamic acid matrix solution was prepared at 50 × 10^−3^ mol L^−1^ in H_2_O:ACN:TFA (50∶50∶0.3, v:v:v). Protein average mass was obtained in the reactor mode with external calibration, using the Protein Calibration Standard I for mass spectrometry (up to 25.000 Da mass range, Bruker Daltonics, Billerica, MA). In addition, the protein was analyzed by ESI optimization conditions performed by injecting in triplicate a standard solution containing standard calibrants at a concentration of 10 ppm. Protein was analyzed immediately after preparation. The extracted ion chromatogram peak areas obtained for each peptide ion were calculated for the JTI molecular mass. Optimized ESI conditions were ion polarity, positive; nebulizer pressure, 4.4 psi; capillary voltage, 4500 V; gas temperature, 180°C; gas flow, 4 L min^−1^. After the purity and molecular mass analysis of JTI, it was reduced and alkylated, and digested with immobilized porcine pepsin in solution. Also, the fraction corresponding to the inhibitor in SDS‐PAGE (sodium dodecyl sulfate polyacrylamide gel electrophoresis) (see above) was digested in the gel by porcine trypsin. For reduction and alkylation, 50 µg of the purified inhibitor was used. Reduction was performed in 300 µL of 0.1 mol L^−1^ sodium hydrogen carbonate with 0.05 mol L^−1^ 1,4‐dithiotreitol for 60 min at 70°C, followed by alkylation with 0.1 mol L^−1^ 2‐iodoacetamide in a final volume of 600 µL (0.1 mol L^−1^ sodium hydrogen carbonate) for 40 min at 37°C. Samples were then filtered (0,22 µm) and the alkylated protein was purified by reversed‐phase chromatography Vydac analytical C18 column (250 × 0,46 cm), HPLC. Experimental conditions were as follows: H_2_O:ACN:TFA (95∶5∶0.1, v:v:v) for 5 min, then a linear gradient to H_2_O:ACN:TFA (5∶95∶0.1, v:v:v) over 65 min; 1 mL.min^−1^ flow rate; 40°C; 250 µL of the solution injected. Pepsin hydrolysis was conducted for 12 h in 100 m mol L^−1^ sodium acetate buffer, pH 4.5, with immobilized enzyme. The obtained peptides were purified by narrow‐bore reversed‐phase chromatography. Separation was performed in a 30 × 2 mm Model XR‐ODS column (Shimadzu, Kyoto, Japan) in the UFLC system. Experimental conditions were as follows: H_2_O:ACN:TFA (95∶5∶0.1, v:v:v) for 5 min, then a linear gradient to H_2_O:ACN:TFA (5∶95∶0.1, v:v:v) over 30 min; 0.4 mL min^−1^ flow rate; 40°C; 50 µL of the peptide solution injected. For trypsin digestion, the gel containing the protein band was excised and transferred to a microfuge tube. The dye Coomassie Blue was taken from the gel with three washes made with aqueous 30% ethanol (by volume) to bleach completely, followed by another wash with aqueous 50% ACN and 2.5 × 10^−2^ mol L^−1^ ammonium carbonate for 15 min. ACN was added, and the mixture was allowed to incubate for 10 min under vigorous stirring. After this step, the gel was dried at reduced pressure (Centri‐Vap, Labconco, Kansas City, USA) for 20 min. To the dried gel pieces it was added a solution of trypsin (33 ng µL^−1^) in a volume sufficient to cover the gel, and these suspensions were maintained in an ice bath for 30 min. A 40 µL volume of a solution of ammonium carbonate (5.0 × 10^−2^ mol L^−1^) was then added, and the system was incubated for 19 h at 37°C [51]. For molecular mass analysis, α‐cyano‐4‐hydroxycinnamic acid (CHCA) at 50 × 10^−3^ mol L^−1^ in 0.3% aqueous acetonitrile was employed as a matrix. The peptides obtained by JTI hydrolysis with pepsin and trypsin were mixed with CHCA in a proportion of 1:3 (v:v) and deposited onto an AnchorChip target (Bruker Daltonics, Bilerica, USA) and allowed to crystallize at room temperature. The ionization was performed in the positive reflected mode, with the following instrument voltage parameters: Ion source 1: 20.00 kV, ion source 2: 17.65 kV, lens: 7.50 kV, reflector: 22.00 kV, reflector 2: 9.80 kV. Data were recorded in the *m/z* range from 600 to 4,000. Peptide fragmentation was conducted by the LIFTM methodology [52] with the following instrument voltage parameters: Ion source 1: 6.00 kV, ion source 2: 5.25 kV, lens: 3.00 kV, reflector 1: 27.00 kV, reflector 2: 11.80 kV, LIFT 1, 19.00 kV, LIFT 2: 4.70 kV [52]. The spectra interpretation and peptide sequencing were manually performed by using the FlexAnalysis 3.3 software (Bruker Daltonics, Bilerica, USA).

### 5.8 Hemolytic and Cytotoxic Activity of JTI

Human heparin blood was obtained from bank blood donations and stored at 4°C. Collection was obtained with written informed consent. The evaluation of hemolytic activity was performed according to Johansson et al. [53]. Triton X‐100, 1% (v:v) was the reference for 100% hemolysis. PBS (Phosphate‐buffered saline) buffer at 0.15 mol L^−1^, pH 7.4 was the reference for 0% hemolysis. The experiment was conducted with the approval of the CEUA under number 014/2018.

### 5.9 Cytotoxic Assays

Cytotoxic assays were performed to evaluate JTI effects on transformed (B16‐F10 cells) and normal cell lines (3T3 and Raec cell lines). Cells were treated with varying concentrations of the purified protease inhibitor and assessed using the MTT (3‐(4,5‐dimethylthiazol‐2‐yl)‐2,5‐diphenyltetrazolium bromide) colorimetric assay [16]. Cells were dispensed in 96‐well flat‐bottomed microtiter plates (TPP products, Switzerland) at a density of 5 × 10^3^ cells.well^−1^ and then incubated for 24, 48, and 72 h with JTI at 0.003 to 1 µM. The test was performed in triplicate. The measurement of cell proliferation inhibition was carried out in comparison with a control containing untreated cells with JTI as follows:

$$\mathrm{Inhibition}\;\mathrm{rate}=\frac{\left({\mathrm{Abs}}_{570}\;\mathrm{control}\;-\;{\mathrm{Abs}}_{570}\;\mathrm{sample}\right)}{{\mathrm{Abs}}_{570}\;\mathrm{control}}\;\times 100$$

### 5.10 Viability Assays

An Alamar Blue assay was performed to analyze the effects of JTI on Raec viability. Cells were cultured (10^4^ cells well^−1^) in 96 well plates and, after synchronization and application of the inhibitor (0.325, 0.65, and 1.3 µM) for 24, 48, and 72 h of exposure, the resazurin salt (44 µM) was then applied and incubated for 2 to 4 h for further analysis with a fluorescence reader at 590 nm. BrdU assay was also performed to visualize JTI effects on B16‐F10 cells.

### 5.11 Annexin V‐FITC/PI Double Staining and Analysis by Flow Cytometry

To evaluate the effects of JTI on cell death, the FITC/annexin V Apoptosis Kit with Dead Cell Annexin FITC (fluorescein isothiocyanate) and PI (propidium iodide), for flow cytometry (Invitrogen, Catalog No. V13242) was used. Cells were grown in 6‐well plates until they reached a confluence of 2 × 10^5^ cells mL^−1^ with medium without serum and stimulated to exit G_0_ in the presence of purified lectin solubilized in DMEM, supplemented with 10% FBS for 24, 48, and 72 h. In addition, a negative control was prepared without the presence of JTI. After exposure to a concentration of 0.65 µM JTI, B16‐F10 cells were trypsinized, collected, and washed with cold PBS. The supernatant was discarded, and the cells were resuspended in 200 µL of 1X binding buffer. 5 µL of Annexin V‐FITC and 1 µL of PI solution (100 µg mL^−1^) were added to a 100 µL cell suspension. The cells were incubated for 15 min at room temperature and kept under light protection. After the incubation period, 250 µL of binding buffer for Annexin V 1X was added, and cells were analyzed by flow cytometry (flow cytometer FASCANTO II, BD Biosciences), measuring the fluorescence emission at 530–575 nm for Annexin V and 630–22 nm for PI. The population was divided into four groups: viable cells (low fluorescence levels), cells in the early stages of apoptosis (green fluorescence), cells in the final stages of apoptosis (green and red fluorescence), and cells in necrosis (red fluorescence). The percentage of cells undergoing apoptosis was determined every 20,000 events, and the graphs obtained in the experiment represent data from three independent experiments. For data analysis, FlowJo software (Tree Star, Inc., CA, USA) was used.

### 5.12 Cell Cycle Assay

B16‐F10 cells were washed with cold PBS, and the supernatant was discarded. The pellet with cells was then incubated with 2% paraformaldehyde, washed with cold PBS, and permeabilized with 0.01% saponin for 15 min. After this procedure, the cells were incubated with 10 µL of RNase (4 mg mL^−1^) at 37°C for 30 min. 5 µL of PI solution (25 mg mL^−1^), along with 200 µL of cold PBS, was added to the cells, which were added and taken to the flow cytometer for analysis of cell cycle arrest (630–22 nm). The percentage of apoptotic cells was determined every 20,000 events, and graphs obtained in the experiment represent data from three independent experiments. FlowJo software (Tree Star, Inc., CA, USA) was used for data analysis.

### 5.13 Mitochondrial Membrane Potential Assay

Changes in mitochondrial membrane potential (ΔΨm) were evaluated and analyzed by flow cytometry in cells after incubation with JTI, according to the method previously described in the literature [54]. After trypsinization, cells were collected and washed twice with PBS, and then the concentration of the cell suspension was adjusted to 1 × 10^6^ cells mL^−1^. A volume of 100 µL solution of Rhodamine 123 (Rh123) (505/535 nm) (20 µg mL^−1^) was added to the collected cells and incubated at 37°C in the dark for 30 min. Subsequently, the cells were washed again with PBS and stained with propidium iodide (PI) solution (100 µg mL^−1^), washed with phosphate‐buffered saline twice, and prepared for analysis in the flow cytometer (FACSCanto, BD Bioscience). All data were collected, stored, and analyzed with the software FlowJo v. 7.6.3 (Tree Star, Inc., CA, USA).

### 5.14 Immunofluorescence

For cytochemical detections, tumor B16‐F10 cells were seeded on circular coverslips 12 mm in diameter at a concentration of 10^4^ cells:coverslip and kept in a 24‐well plate. The experiments were performed with cells cultured for 3 days. Then the medium was removed, and cells were incubated with fresh medium in the presence or absence of 0.65 µM of JTI. After the required period, the medium was removed, cells were washed five times in PBS (0.1 M, pH 7.4) at 4°C, and the assay was then performed. The cells were incubated with various markers [WGA lectin conjugated to FITC (5 mg mL^−1^) (W11262‐ Lot 997854); Phalloidin (A12379); COX ‐ 4 (48505); Β‐actin; Β‐tubulin; VEGF (Vascular endothelial growth factor); Vimentin (SC‐73259); Fibronectin (CS‐6953); Caspase‐3 (# 9664S); Chondroitin (370615); IL‐6 (SC‐130326); Mitotracker Red (M22425)]. These markers were applied to the cells in PBS for 1 h at 4°C. Next, the cells were washed five times with PBS and fixed with 2% formaldehyde in PBS for 30 min at 25°C. Subsequently, the cells were sequentially washed in PBS two times, one time in PBS containing 0.1 M glycine, and two times in PBS. After appropriate markings, cells were permeabilized using a solution of PBS containing 0.01% saponin. Then the coverslips were washed five times in PBS, washed again using double‐distilled water, and mounted on slides using Prolong mounting medium containing DAPI antifade gold, which allowed the color of the core. The images were obtained on a confocal microscope, Leica SP8.

### 5.15 Reactive Nitrogen Species (RNS) and Reactive Oxygen Species (ROS) Assays

Melanoma cell line (B16‐F10, ATCC) was cultured in sterile disposable Petri dishes (35 × 10 mm) on glass coverslips at a ratio of 3 × 10^4^ cells:coverslip, using DMEM medium supplemented with 10% fetal bovine serum at 37°C under a humid atmosphere of 5% CO_2_. After synchronization and incubation with trypsin inhibitor (0.65 µM), the cells were analyzed, and the effect of the inhibitor on the release of nitric oxide and total free radicals was evaluated at times of 30 min, 6 h, and 72 h of exposure by fluorescence microscopy (Leica CTR HS—TIRF). For testing release and quantification of nitric oxide, DAF‐FM diacetate reagent (4‐amino‐5‐methylamino‐2',7'‐difluorofluorescein diacetate) (Invitrogen, Life Technologies) at 10 µM was used in HBSS (Hank's balanced solution). The probe was incubated for 30 min in the incubator at 37°C in a humidified atmosphere of 5% CO_2,_ and after the incubation time, the cells were observed under a fluorescence microscope. For the assay quantification of reactive oxygen species (ROS), the probe H2DCFDA (Invitrogen, Life Technologies) at 5 µM was used in HBSS. The probe was incubated for 30 min in an incubator at 37°C in a humidified atmosphere of 5% CO_2,_ and after the incubation time, the cells were observed under a fluorescence microscope.

### 5.16 Cytosolic Calcium Release Assay

Melanoma cells were cultured in 96‐well plates (1 × 10^4^ cells per well) for three days, and cytoplasmic calcium was then determined using the Fluo4 Direct Kit [55]. For this purpose, the plates containing cells and culture medium were incubated for 1 h at 37°C with a solution of Direct Fluo4. JTI solution was added to the plate at a final concentration of 0.65 µM in 20 s, and fluorescence was monitored for at least 300 s. The experiment was carried out in a microplate reader at 37°C.

### 5.17 Western Blotting Assay

B16‐F10 cells were plated at a concentration of 9.6 × 10^5^ cells in 75 mL sterile bottles and incubated for 24 h for adhesion. A fixed concentration of 0.65 µM of JTI was added to cells at different times of incubation (0, 24, 36, 48, and 72 h), washed with cold PBS and removed with 200 µL of lysis buffer [0.05 M tris‐HCl (pH 7.4), 1% Tween 20, 0.25% sodium deoxycholate, 0.15 M NaCl, 1 mM EGTA, 1 mM Na_3_VO_4_, 1 mM NaF and the following protease inhibitors for 2 h on ice: 1 µg mL^−1^ aprotinin, 10 µg mL^−1^ and 1 mM leupeptin fluoride of phenylmethanesulfonyl]. Total protein extracts were obtained, and a polyacrylamide gel electrophoresis in the presence of SDS was carried out following an established methodology [48]. Protein extracts were resolved and electrophoretically transferred to a PVDF (polyvinylidene fluoride) membrane (Millipore, Bedford, MA, USA). After transfer, membranes were blocked for non‐specific sites with blocking buffer [1% skim milk or 2% BSA in TBS (Tris‐buffered saline) with 0.05% Tween 20 (TBST)], remaining in this solution for 1 h, and then incubated for about 12 h at 4°C with appropriate primary antibody diluted in blocking buffer at a ratio of 1:1000. After washing in TBST, membranes were incubated with anti‐rabbit secondary antibody conjugated with peroxidase, diluted 1:2000 in blocking buffer for 1 h. The detection was performed using chemiluminescence [56].

### 5.18 Cell Migration Assay

To evaluate the stimulation of cell migration due to JTI action, a cell migration assay was performed. The B16‐F10 tumor cells were cultured in 24‐well plates (Corning Inc.). After cell adhesion and proliferation until the confluence state, a monolayer favorable to the development of the assay, the culture medium was replaced by DMEM supplemented with fetal calf serum, and a diametral risk was made using a sterile pipette tip P‐1000 (Axygen) in each well. The plates were washed to remove possible loose cells, and then different concentrations of cells were applied to JTI (0.325, 0.65, and 1.3 µM), where the effect of the inhibitor was evaluated in the following exposure times: 0, 3, 6, 18, and 24 h. Cells without treatment with the inhibitor were used as controls to evaluate cell migration potential. After each exposure time, the cells were observed in phase contrast microscopy and photographed using a camera (Sony Cyber‐Shot). The distance of cell migration was determined by measuring the width of the “wound”, subtracting half the value of the initial width at half the height of the wound. The average wound width can be obtained from the wound area by dividing the area by the length of the analyzed region. The widths of the wounds obtained were plotted against time using Microsoft Excel (Redmond, WA), and a linear fit was generated for each data set. The slope of the linear fit was used as a measure of cell migration. The migration was expressed as a percentage of relative wound closure in relation to the initial wound area at 0 h. Data were normalized to the untreated control group to account for basal migration rates. Each experiment was performed in triplicate.

### 5.19 Angiogenesis Assay

96‐well plates were preincubated with 50 µL of reconstituted basement membrane (10 to 15 mg mL^−1^ protein) at 37°C in a 2.5% CO_2_ atmosphere for 3 h. After this period, 5 × 10^4^ cells well^−1^ were plated in the presence of 200 µL of F12 medium containing 10% FBS and incubated for 12 h at 37°C with 2.5% CO_2_. Cells were observed and analyzed using an inverted optical microscope in phase contrast. The results were expressed based on the quantification of the number of formed vessels and the length thereof. Matrigel used in this experiment was produced by the laboratory, and the purification of protein EHS tumor matrix (Engelbreth–Holm–Swarm) for the preparation of matrigel was performed according to the method described by Kleinman [57].

### 5.20 Statistical Analysis

Data are expressed as the mean ± standard deviation (SD) of at least three independent experiments performed in triplicate. Significant differences were considered when the *p*‐value was equal to or less than 0.05. We used the parametric analysis test, analysis of variance (ANOVA), followed by the Tukey test (significance level of *p* ≤ 0.05) in the software IBM SPSS STATISTIC 20.

## Authors Contributions

**Luciana Maria Araújo Rabêlo**: conceptualization, writing and original draft preparation, investigation, methodology, and formal analysis. **Leonardo Thiago Duarte Barreto Nobre**: investigation, methodology and formal analysis. **Paula Ivani Medeiros dos Santos**: investigation, methodology and formal analysis. **Sheyla Varela Lucena**: investigation, methodology, and formal analysis. **Raphael Paschoal Serquiz**: investigation, methodology, and formal analysis. **Hugo Alexandre de Oliveira Rocha**: formal analysis. **Helena Bonciani Nader**: methodology and formal analysis. **Marcelo Porto Bemquerer**: methodology and formal analysis. **Elizeu Antunes dos Santos**: review and editing. **Adeliana Silva de Oliveira**: review and editing. **Breno Emanuel Farias Frihling**: data curation, writing, review, and editing. **Pedro Henrique de Oliveira Cardoso**: data curation, writing, review and editing. **Ludovico Migliolo**: data curation, writing, review and editing. All authors reviewed the results and approved the final version of the manuscript.

## Funding

This work was supported by the Brazilian funding agencies CNPq, CAPES, and FUNDECT.

## Conflicts of Interest

The authors declare no conflicts of interest.

## Supporting information




**Supporting File**: cbdv71227‐sup‐0001‐SuppMat.docx.

## Data Availability

All data for this research are contained within the manuscript, for further clarification if needed contact the corresponding author.
