# Supplementary material for: Biochemical Characterization of a Kunitz‐Type Protease Inhibitor From Mimosa regnellii and Its Effects on Melanoma Cell Viability and Angiogenesis
Source: Chem Biodivers. 2026 Apr 21;23:e03760. doi: 10.1002/cbdv.202503760 (PMC13098310; doi:10.1002/cbdv.202503760)
Supplement: Supplementary file 1 — Supporting File: cbdv71227‐sup‐0001‐SuppMat.docx. [file CBDV-23-e03760-s001.docx]

Supporting Information

Biochemical Characterization of a Kunitz-Type Protease Inhibitor from *Mimosa regnellii* and its Effects on Melanoma Cell Viability and Angiogenesis

Luciana Maria Araújo Rabêlo^‡^, Pedro Henrique de Oliveira Cardoso^‡^, Leonardo Thiago Duarte Barreto Nobre, Paula Ivani Medeiros dos Santos, Sheyla Varela Lucena, Raphael Paschoal Serquiz, Marcelo Porto Bemquerer, Hugo Alexandre de Oliveira Rocha, Helena Bonciani Nader, Elizeu Antunes dos Santos, Adeliana Silva de Oliveira, Breno Emanuel Farias Frihling, Ludovico Migliolo*


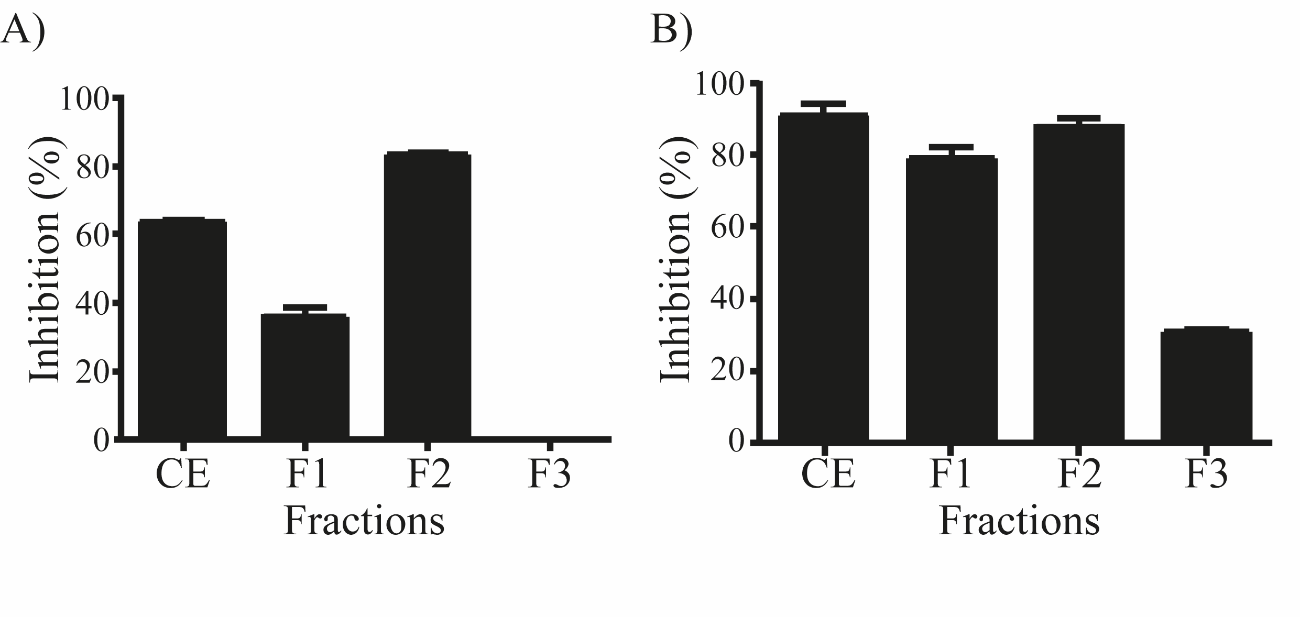


**Figure S1 – *M. regnellii* crude extract and purified fractions enzymatic inhibition assay against the serine proteases trypsin using 1% azocasein.**


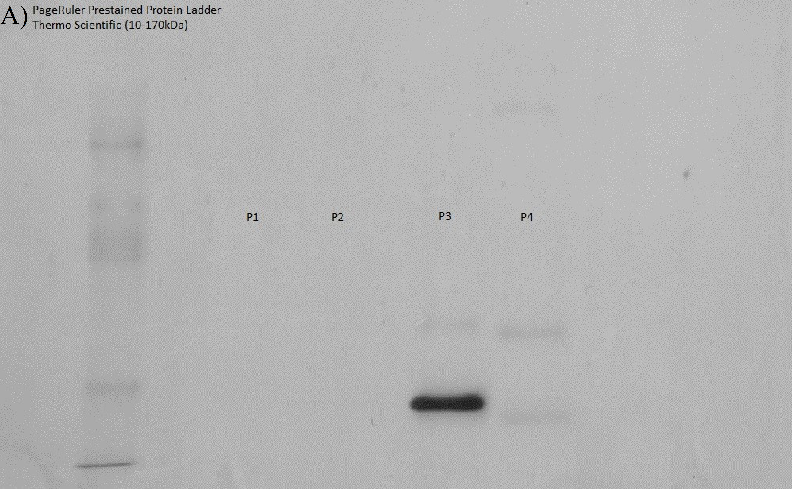

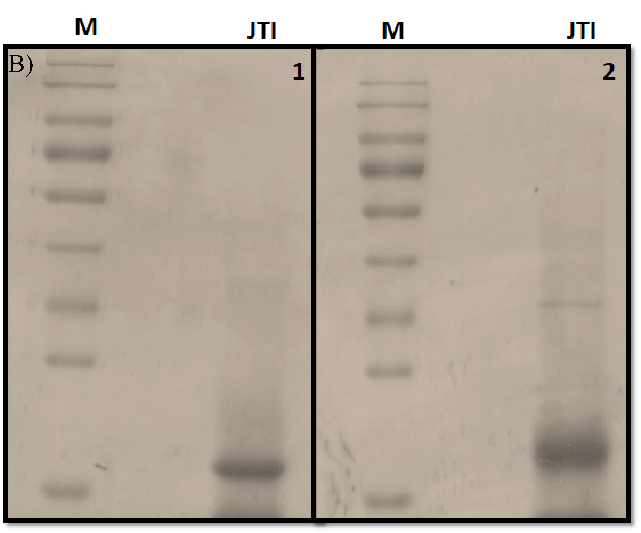


**Figure S2 - A) SDS-PAGE Electrophoresis of fractions from the RP-HPLC. Gel (15% polyacrylamide SDS) was revealed using the protocol for staining with silver nitrate solution. Molecular weight marker [PageRuler prestained protein ladder (10 – 170 kDa) Cat # SM0671]; P1 - 1 peak, retention time of 3 sec.; P2 - peak 2, retention time 12 sec.; P3 - 3 peak, retention time 22 sec.; P4 - peak 4 of 24 sec retention time. B) - Electrophoresis JTI denaturing and reducing conditions. 1) Gel undergoing treatment with 10% SDS; 2) Gel undergoing treatment with 25 mM DTT. Stained gel using the protocol for labeling with Coomassie blue solution. Molecular weight marker [PageRuler prestained protein ladder (10 – 170 kDa) Cat # SM0671]; JTI - Juquiri Trypsin Inhibitor.**


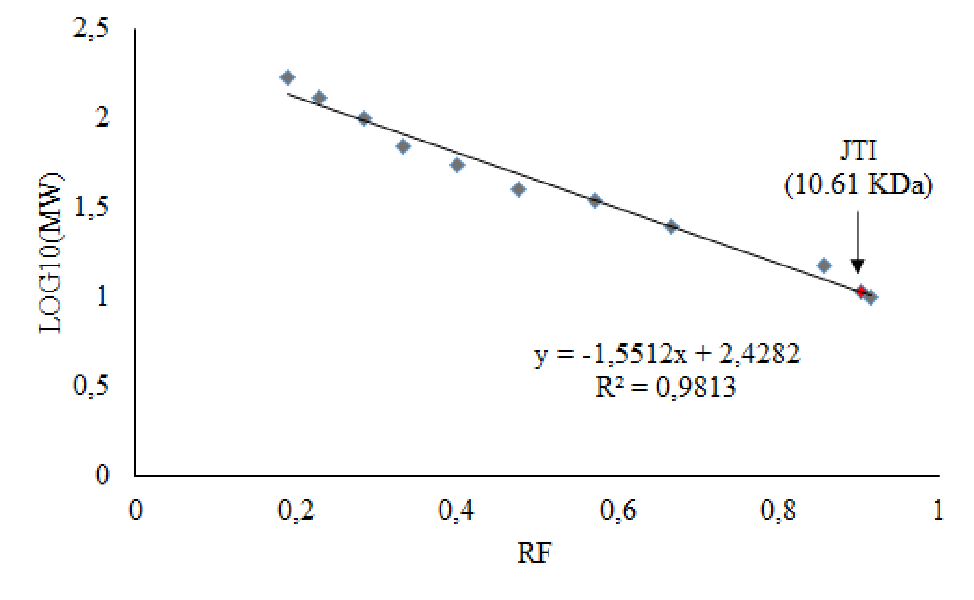


**Figure S3 - Determination of the apparent molecular mass of Juquiri Trypsin Inhibitor (JTI) by electrophoresis. The molecular weight JTI (~ 10.61 kDa) was determined by polyacrylamide gel electrophoresis in SDS. The molecular weight marker used was PageRuler prestained protein ladder (10-170 kDa, Cat # SM0671). Proteins were stained with silver nitrate. Mr log - log of molecular weight.**

A)

B)

C)

**Figure S4 – *De novo* sequencing by MALDI-ToF mass spectrometry of trypsin-digested JTI. A) Fragment 1, B) Fragment 2 and C) Fragment 3.**

**Table S1. The N-terminal partial sequence of JTI with multiple alignments of known plant inhibitors. After sequencing JTI by trypsinization a mass spectrometry was carried out using the BLASTP alignment tool available at the NCBI website (http://blast.ncbi.nlm.nih.gov/Blast.cgi?PROGRAM=blastp&PAGE_TYPE=BlastSearch&LINK_LOC= blasthome). (*) Represent conserved amino acid, (:) represent strong amino acid similarity, (.) represent weak amino acid similarity.**

| **Name** | **Alignment** |
| --- | --- |
| **PjTI** | QELLDVDGEILRNGGSYYILPAFRGKGGGLELAKTEGETCPLTVVQAR |
| **JTI**  **Trypsinized**  **fragments** | Fragment 1 Fragment 2 Fragment 3 -  **PLVLDSDGEPLDGGGPYYILPVH--KGGGLEVAKTGTETCPLSVVQAR**  **:** *** * .** *****.. ******:*** *****:******* |

JTI: Juquiri trypsin inhibitor; PjTI: *Prosopis juliflora* Kunitz-type Trypsin Inhibitor.


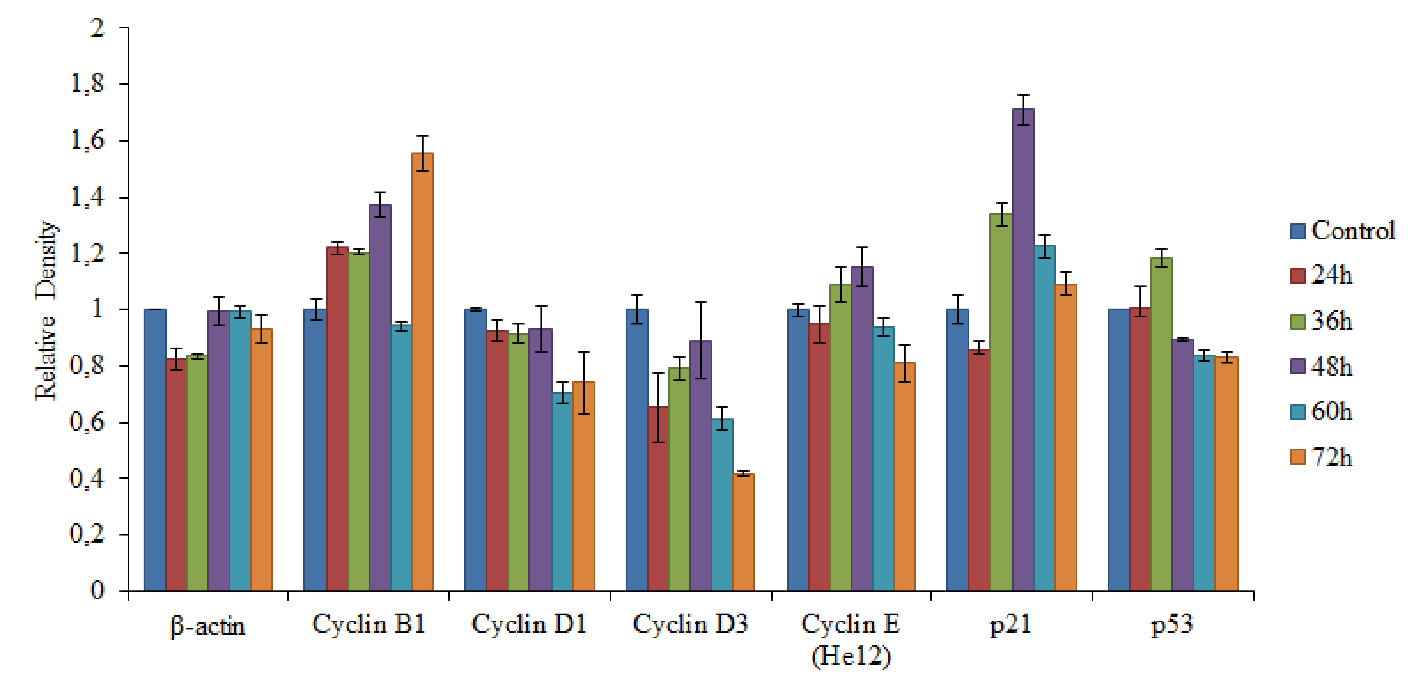


**Figure S5 - Protein expression involved in cell cycle arrest of B16-F10 cells under JTI action. Data obtained from the densitometry of Western Blotting of the protein’s cyclin B1, cyclin D1, cyclin D3, cyclin E, p21, and p53 in melanoma cells (B16-F10) after exposure to JTI at 24, 36, 48, 60 and 72 h.**


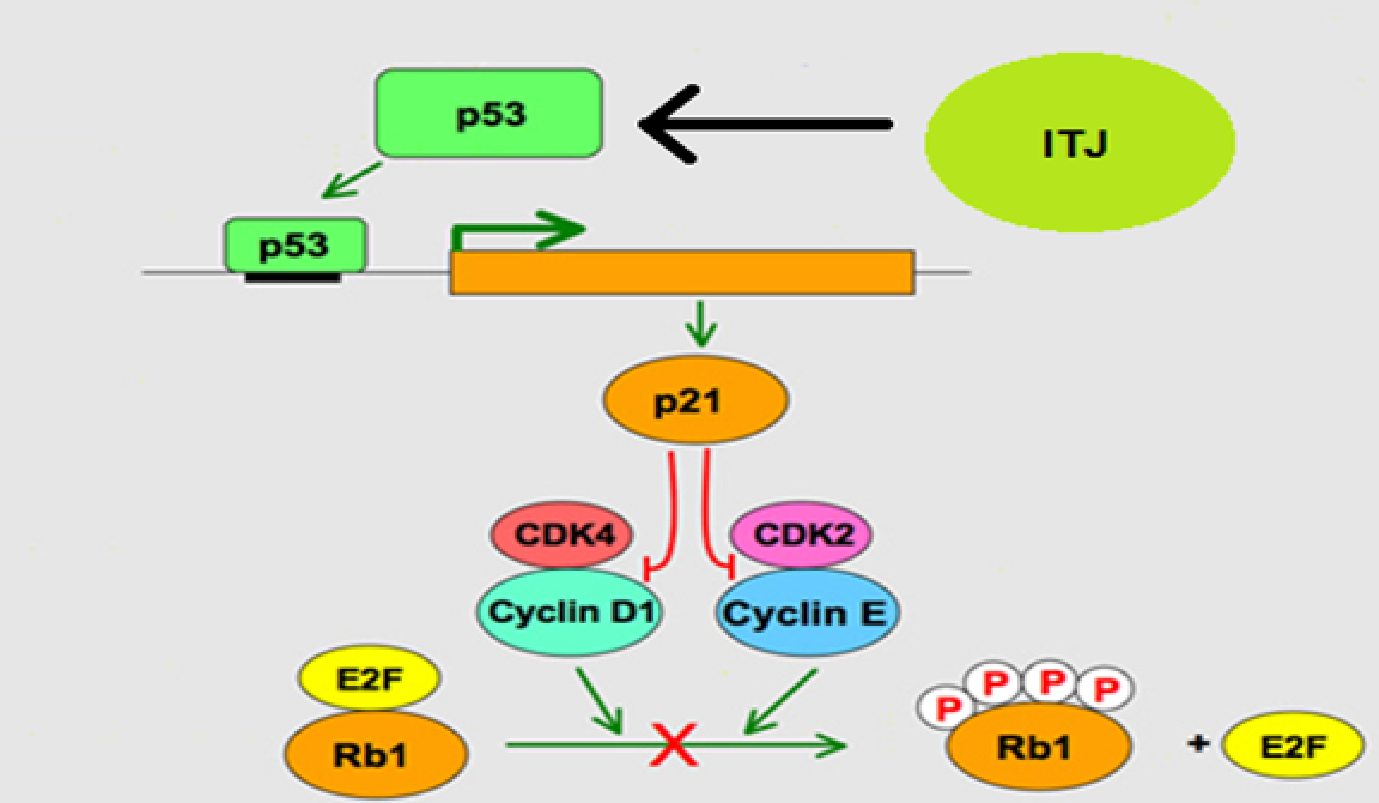


**Figure S6 - JTI possible mechanism of action of the activation of proteins related to cell cycle arrest in B16-F10 melanoma cells. RB - Retinoblastoma protein; P - phosphate. (Modified figure:** [**http://p53.free.fr/p53_info/p53_Pathways.html**](http://p53.free.fr/p53_info/p53_Pathways.html)**).**


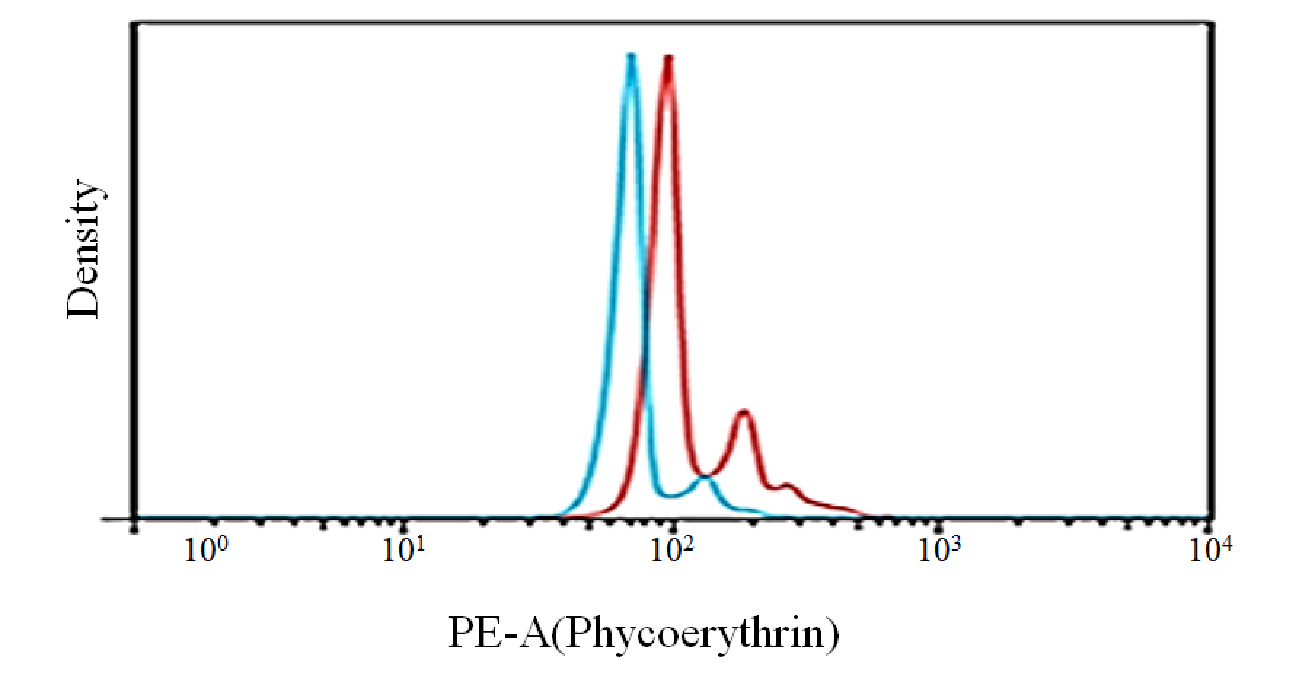


**Figure S7 - Flow cytometric analysis of JTI effect on the mitochondrial membrane potential of B16-F10 cells. Representative histogram of cell populations acquired by FlowJo software v. 7.6.3 (Tree Star, Inc., CA, USA) of B16-F10 strain of tumor cells labeled with rhodamine fluorescence probe 123. Red Line - control group without treatment JTI; Blue line - treated group JTI at a concentration of 0.65 µM, corresponding to the IC_50_.**


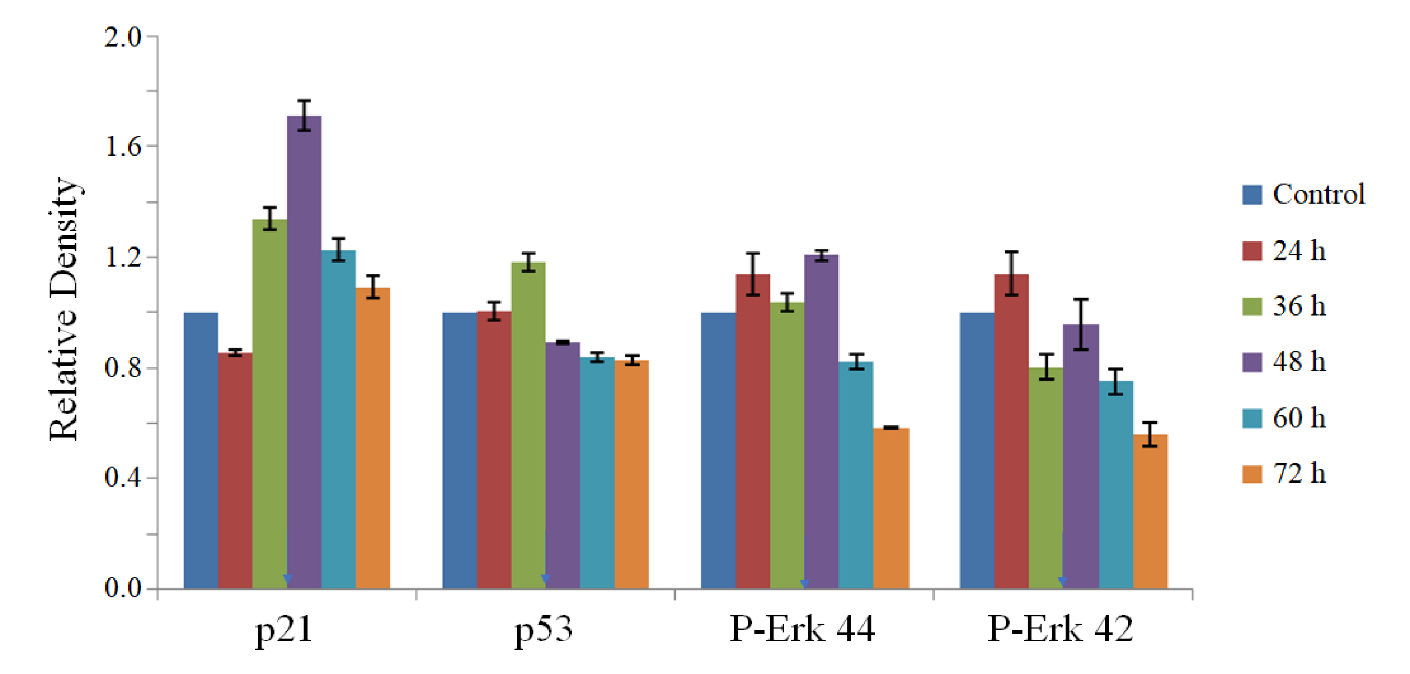


**Figure S8 - Immunoblotting of proteins involved in cell death by apoptosis (p21, p53, p-Erk) B16-F10 cells exposed to JTI. Densitometric Data from the Western Blotting of proteins p21, p53, p-Erk expressed on melanoma cells (B16-F10) after exposure to JTI at 24, 36, 48, 60, and 72 h.**
